# Do Adjunctive Therapies with Natural Products Improve Periodontal Clinical Parameters After Non-Surgical Treatment? A Systematic Review and Meta-Analysis

**DOI:** 10.3390/ijms27052394

**Published:** 2026-03-04

**Authors:** Rafael Scaf de Molon, Joao Victor Soares Rodrigues, Erica Dorigatti de Avila, Davi da Silva Barbirato, Joao Pedro Franco Moura, Gabriele Vanzela Monteiro, Marcos Vinicius Alves, Leticia Helena Theodoro, Rolando Vernal, Wim Teughels

**Affiliations:** 1 Department of Diagnostic and Surgery, School of Dentistry, São Paulo State University (UNESP), Araçatuba 16015-050, São Paulo, Brazil; joao.vic.t@hotmail.com (J.V.S.R.); erica.avila@unesp.br (E.D.d.A.); jp.moura@unesp.br (J.P.F.M.); gv.monteiro@unesp.br (G.V.M.); marcos.v.alves@unesp.br (M.V.A.); leticia.theodoro@unesp.br (L.H.T.); 2 Dental School, Pernambuco Faculty of Health (FPS—Faculdade Pernambucana de Saúde), Recife 51180-001, Pernambuco, Brazil; davibarbirato@gmail.com; 3 Periodontal Biology Laboratory, Department of Conservative Dentistry, Faculty of Dentistry, Universidad de Chile, Santiago 8380492, Chile; rvernal@uchile.cl; 4 Department of Oral Health Sciences, KU Leuven & Dentistry (Periodontology), University Hospitals Leuven, 3000 Leuven, Belgium; wim.teughels@uzleuven.be; 5 Department of Periodontology, Faculty of Dentistry, Chulalongkorn University, Bangkok 10330, Thailand; 6 Department of Restorative Dentistry, Faculty of Dentistry, University Malaya, Kuala Lumpur 50603, Malaysia

**Keywords:** periodontitis, adjunctive therapies, pathogenesis, periodontal disease, alveolar bone, etiology

## Abstract

Periodontitis is a highly prevalent chronic inflammatory disease initiated by dysbiotic biofilms and sustained by an exaggerated host immune response, for which scaling and root planing (SRP) remains the cornerstone of therapy. However, mechanical debridement alone may be insufficient to fully resolve inflammation in complex cases and in susceptible patients. In this context, natural products and host modulatory strategies have emerged as potential adjunctive therapies owing to their antimicrobial, anti-inflammatory, antioxidant, and immunomodulatory properties. This systematic review and meta-analysis aimed to evaluate the effectiveness of natural products used as adjuncts to SRP on periodontal clinical outcomes. Comprehensive electronic searches were conducted in MEDLINE/PubMed, Scopus, Web of Science, Cochrane CENTRAL, Embase, SciELO, and Google Scholar through December 2025, and randomized controlled clinical trials were included. Ninety studies were eligible for qualitative synthesis, and thirty-three were incorporated into the meta-analysis. The interventions encompassed a broad spectrum of plant-derived, host-modulatory and nutraceutical compounds, including curcumin, resveratrol, propolis, *Aloe vera*, green tea catechins, and omega-3 fatty acids, administered via local, systemic, or rinse-based approaches. Meta-analytic findings demonstrated that adjunctive natural products significantly enhanced probing pocket depth (PPD) reduction and clinical attachment level (CAL) gain compared with SRP alone, with additional improvements in gingival inflammation and bleeding outcomes; however, substantial heterogeneity was observed among studies. Overall, natural products provide measurable adjunctive benefits to SRP in the management of periodontitis, although further well-designed, standardized, and long-term randomized trials are necessary to support their routine clinical implementation.

## 1. Introduction

Periodontitis is a chronic, multifactorial inflammatory disease characterized by progressive destruction of the tooth-supporting tissues [1,2,3,4,5,6,7]. It arises from a dysbiotic shift in the subgingival microbiome that triggers a sustained host immune-inflammatory response. Although microbial colonization initiates disease, tissue breakdown is largely driven by an imbalance between pathogenic biofilms and host defense mechanisms. This dysregulated inflammatory cascade promotes connective tissue degradation and alveolar bone resorption, ultimately leading to tooth loss if untreated [1,2,3,4,5,6,7]. Periodontal pathogens such as *Porphyromonas gingivalis*, *Tannerella forsythia*, and *Treponema denticola* contribute to disease progression by activating host immune signaling pathways and amplifying local inflammatory mediators, which together sustain tissue destruction and impair resolution [8,9,10].

Epidemiologically, periodontitis is among the most prevalent chronic diseases worldwide, affecting nearly half of adults and over 10% with severe forms, making it the sixth most common human disease [11,12,13]. Its burden increases with age and is amplified by systemic conditions, behavioral risk factors, and socioeconomic disparities. Beyond localized tissue destruction, periodontitis reflects a dysregulated host inflammatory response that influences both disease progression and treatment outcomes. Persistent inflammation and oxidative stress may limit the effectiveness of mechanical debridement alone, highlighting the importance of therapeutic strategies that extend beyond biofilm disruption [4,14,15]. This host-driven component provides a clinical rationale for adjunctive approaches aimed at modulating inflammation and improving tissue response during non-surgical periodontal therapy (NSPT) [2,3,4,6,7,16,17,18,19]

In this clinical context, contemporary management of periodontitis is guided by evidence-based recommendations from the European Federation of Periodontology, which advocate a stepwise, personalized approach to disease control and prevention [20]. Scaling and root planing (SRP) remains the cornerstone of NSPT, effectively reducing microbial burden and inflammation. However, SRP alone does not always achieve complete clinical stability. Anatomical complexity, persistent biofilm reservoirs, and host-related factors, particularly in patients with systemic conditions may limit treatment responsiveness and sustain inflammatory activity [20,21,22]. These limitations underscore the need for adjunctive strategies that enhance therapeutic outcomes beyond SRP alone.

To address the limitations of SRP, adjunctive strategies have increasingly been incorporated into periodontal care [2,3,4,7,17,23,24]. Current clinical guidelines recognize a range of adjunctive modalities, including locally or systemically administered antimicrobials [16,25], host-modulation agents [26,27,28,29], probiotics and postbiotics [30,31,32,33,34,35,36,37], photodynamic therapy (aPDT) [38,39,40,41], and biologically active molecules such as specialized pro-resolving mediators (SPMs) [6,42,43,44,45,46,47] and small anti-inflammatory compounds [6,42,43,44,45,46,47]. While adjunctive antimicrobials can enhance short-term clinical outcomes, their broader use is constrained by concerns related to resistance, safety, patient adherence, and cost [25,48,49,50]. These limitations have stimulated growing interest in alternative adjunctive approaches that are biologically compatible and suitable for repeated use. In this context, natural products have emerged as promising candidates, offering multifunctional properties that may complement NSPT.

Natural products derived from plant and other biological sources have gained increasing attention as adjunctive therapies in chronic inflammatory conditions, including periodontitis [18,23,24,51,52,53,54,55,56]. Many of these compounds exhibit combined antimicrobial, anti-inflammatory, antioxidant, and host-modulatory properties that are conceptually aligned with the multifactorial pathogenesis of periodontal disease. Representative agents such as curcumin, resveratrol, green tea catechins, *Aloe vera*, and propolis have shown potential to reduce inflammatory burden and support tissue response when used alongside SRP [23,24,51,52,54]. Rather than acting through a single pathway, these agents influence multiple biological processes, including immunemodulation, inflammatory signaling modulation, and oxidative stress regulation, which together may enhance periodontal healing [57,58,59,60,61,62,63,64,65,66,67,68]. Importantly, natural products are often viewed as biologically compatible adjuncts with a favorable safety profile, making them attractive candidates for repeated or maintenance use. However, variability in formulation, dosing, and delivery systems, as well as limited pharmacokinetic standardization, present challenges for clinical translation [17]. These factors underscore the need for rigorous synthesis of current evidence to clarify their therapeutic relevance and inform future research directions.

In recent years, numerous randomized controlled clinical trials have evaluated a wide array of natural products, ranging from topical gels, mouthrinses, and nanofiber scaffolds to systemic supplements, as adjuncts to SRP. Emerging formulations such as nanocarriers, hydrogels, and biodegradable chips have further enhanced the bioavailability and sustained release of active compounds within the periodontal pocket [17]. The cumulative evidence suggests that adjunctive use of natural agents can enhance clinical parameters such as probing pocket depth (PPD) reduction, clinical attachment level (CAL) gain, and decreased bleeding on probing (BOP), while also reducing microbial load and inflammatory biomarkers in gingival crevicular fluid or saliva. Moreover, these agents have shown additional benefits in specific patient populations with systemic comorbidities by modulating systemic inflammation and oxidative stress [69,70,71,72]. Despite these promising findings, heterogeneity in study design, sample sizes, treatment duration, and outcome measures hinders direct comparisons across trials and the establishment of clinical recommendations.

In this review, the term “natural products” refers broadly to biologically derived compounds of plant or other natural origin administered to modulate microbial burden, host inflammatory responses, oxidative stress, or periodontal tissue repair. This umbrella definition includes traditional herbal preparations, phytochemical extracts, and nutraceutical supplements such as omega-3 fatty acids, resveratrol, lycopene, and antioxidant-rich formulations. Although not all agents are strictly herbal, they share a common rationale as non-synthetic adjunctive therapies targeting host–microbe interactions rather than conventional antimicrobial mechanisms. Given the growing and heterogeneous body of evidence, a systematic evaluation is necessary to clarify the therapeutic relevance of these compounds when used alongside SRP. Accordingly, this review critically synthesizes randomized controlled trials to assess clinical, microbiological, and biochemical outcomes, identify knowledge gaps, including the need for standardized formulations and long-term data and inform future translational applications in evidence-based periodontal care.

Despite increasing interest in natural products as adjuncts to NSPT, the evidence base remains fragmented across diverse compounds, delivery systems, and study designs. Previous reviews have primarily focused on narrative summaries or specific agents without integrating quantitative synthesis, methodological quality assessment, and clinical interpretability [73,74,75,76,77,78,79,80,81,82,83]. Moreover, rapid expansion of randomized trials has introduced substantial heterogeneity that complicates translation into practice. A contemporary systematic review and meta-analysis that evaluates clinical outcomes, considers delivery modalities, and incorporates risk-of-bias and certainty-of-evidence frameworks is therefore necessary to clarify the strength, limitations, and clinical relevance of adjunctive natural therapies. Therefore, the objective of this systematic review and meta-analysis is to critically evaluate randomized controlled trials investigating natural products as adjuncts to SRP, with the aim of assessing their clinical, microbiological, and biochemical effects in periodontal therapy.

## 2. Materials and Methods

### 2.1. Protocol Registration and Reporting Standards

This systematic review and meta-analysis were conducted following the methodological standards established by the Cochrane Collaboration [84] and reported in accordance with the Preferred Reporting Items for Systematic Reviews and Meta-Analyses (PRISMA 2020) and the PRISMA-P (Protocols) guidelines [85]. The study protocol was prospectively registered in the International Prospective Register of Systematic Reviews (PROSPERO) under the registration number #CRD420251233170 (https://www.crd.york.ac.uk/PROSPERO/view/CRD420251233170 (accessed on 16 November 2025)). The primary aim of this review was to answer the following question: Do adjunctive therapies with natural products/herbal medicine improve periodontal clinical parameters after SRP?

The research question was formulated according to the PICO framework. The population (P) comprised patients diagnosed with periodontitis. The intervention (I) consisted of SRP combined with the adjunctive use of natural products/herbal medicine or with other pharmaceutical preparations, while the comparison (C) group received SRP alone. The main outcomes of interest were changes in PPD, CAL, and BOP, which represent clinical indicators of periodontal improvement.

### 2.2. Search Strategy

A comprehensive search strategy was designed to identify all potentially relevant studies that addressed the clinical effects of natural products as adjunctive therapies to NSPT. Two reviewers (J.V.S.R. and D.S.B.) independently searched the electronic databases PubMed (http://www.ncbi.nlm.nih.gov/sites/pubmed (accessed on 14 November 2025)) through MEDLINE, Cochrane CENTRAL (https://www.cochranelibrary.com), Web of Science (https://www.webofknowledge.com) through Clarivate Analytics (https://clarivate.com), Embase (https://www.embase.com), and Scopus (http://www.scopus.com) through Elsevier (https://www.elsevier.com). Additional sources such as Scielo.org (https://scielo.org) and bvs|BIREME (https://bvsalud.org) were also searched. Grey literature was explored using Google Scholar (https://scholar.google.com.br (accessed on 14 November 2025)) and OpenGrey’s SIGLE database (www.opengrey.eu) until 14 November 2025.

The search strategy combined controlled vocabulary terms and free-text keywords using the Boolean operators “OR” and “AND”, connecting the key concepts in a “building blocks” strategy. The main terms included are described in Table 1. Each database search strategy was adapted to its specific indexing system and syntax to ensure comprehensive coverage. The electronic searches were performed in November 2025. Databases alerts were set to identify studies published after the time of the searches, until the manuscript submission process.

Reference lists of all included studies and relevant reviews were manually searched for additional publications. A manual search of leading periodontal journals, including the Journal of Clinical Periodontology, Journal of Periodontology, Journal of Periodontal Research and Clinical Oral Investigations, was also conducted to identify articles not indexed in electronic databases.

Although the electronic search strategy primarily employed plant- and herbal-related terminology, additional nutraceutical and non-plant-derived natural compounds were identified through manual screening of references and full-text evaluation, thereby ensuring comprehensive coverage of biologically derived adjunctive therapies.

**Figure 1:**
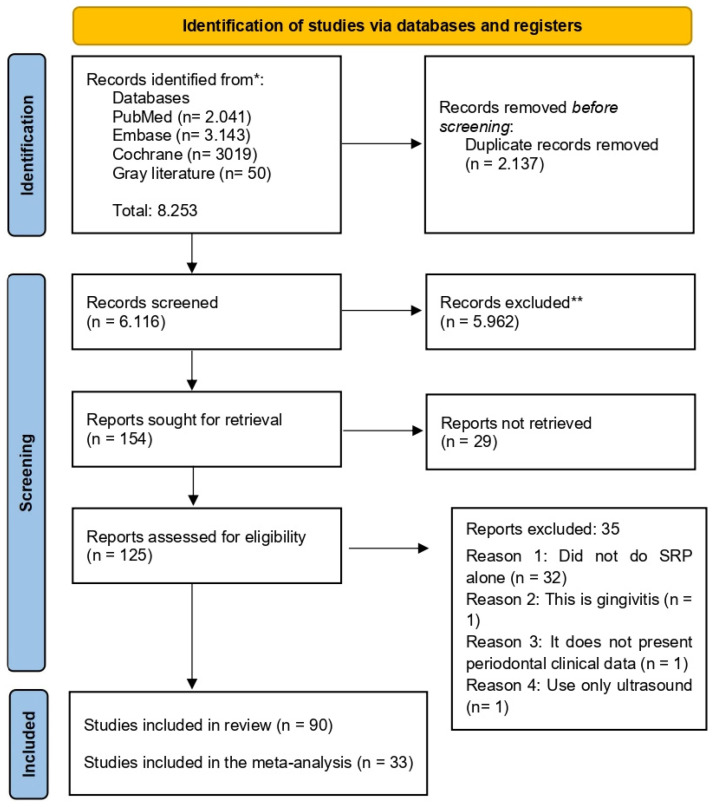
PRISMA flowchart of the included studies [86]. * Consider, if feasible to do so, reporting the number of records identified from each database or register searched (rather than the total number across all databases/registers). ** If automation tools were used, indicate how many records were excluded by a human and how many were excluded by automation tools.

All identified references were exported to Rayyan Reference Manager (https://www.rayyan.ai (accessed on 25 November 2025)), which facilitated record organization and automated duplicate detection. Any remaining duplicates were identified manually. The full selection process was documented in accordance with the PRISMA 2020 flow diagram, detailing the number of studies screened, assessed for eligibility, and included in the final analysis (Figure 1).

### 2.3. Eligibility Criteria

Studies were considered eligible if they were randomized controlled clinical trials evaluating the effects of natural products/herbal medicine as adjuncts to SRP in patients with a clinical diagnosis of periodontitis. Eligible trials were required to include human participants and to report at least one clinical periodontal outcome, such as PPD, CAL, or BOP. Studies that included patients with systemic conditions were accepted, provided that such conditions were evenly distributed between groups.

Publications were excluded if they were not randomized clinical trials, such as case reports, case series, observational studies, reviews, or preclinical studies. Studies that lacked quantifiable clinical periodontal data, were not written in English, or were duplicate reports of the same dataset were also excluded.

### 2.4. Study Selection

The study selection followed a two-stage process to ensure methodological rigor and transparency. In the first stage, titles and abstracts were independently screened by two reviewers (J.V.S.R. and D.S.B.) based on the predefined eligibility criteria. In the second stage, the full texts of all potentially eligible articles were retrieved and examined in detail to confirm inclusion. Disagreements between reviewers at any stage were resolved through discussion, and when consensus could not be reached, a third reviewer (R.S.M.) was consulted to make a final decision.

To ensure consistency and reproducibility, inter-reviewer agreement was calculated using Cohen’s kappa coefficient, which indicates values above 0.85, indicating excellent reliability. This rigorous process ensured that all included studies met the inclusion criteria and that the screening process remained unbiased.

### 2.5. Data Extraction and Management

Data extraction was independently performed by two reviewers (J.V.S.R. and D.S.B.) using a pretested standardized data extraction sheet developed for this review. The extracted information included authorship, year and country of publication, sample size, demographic characteristics of participants, diagnostic criteria for periodontitis, details of interventions and comparators, treatment duration, type and dosage of natural products, mode of administration, follow-up duration, and all reported periodontal clinical outcomes.

Whenever quantitative data were available, the reviewers extracted the mean and standard deviation values for each clinical parameter at baseline and at the latest follow-up, as well as the mean changes over time. When data were incomplete, corresponding authors were contacted for clarification. Information regarding study funding and declared conflicts of interest was also collected to identify potential sources of bias. All extracted data were cross-checked for accuracy, and discrepancies between reviewers were resolved by consensus with the involvement of a third reviewer when necessary (R.S.M.).

Because follow-up durations varied across included trials, outcome data were extracted at the latest reported time point for each study. This strategy was adopted to capture the maximal reported clinical effect and to avoid preferential selection of early outcomes when standardized follow-up intervals were unavailable. While this approach allows inclusion of the most complete dataset from each trial, it introduces temporal heterogeneity, which was considered when interpreting pooled estimates.

### 2.6. Risk of Bias Assessment

The methodological quality of the included randomized controlled trials was assessed using the Cochrane Risk of Bias 2.0 tool (RoB 2) [87]. This tool evaluates potential sources of bias across five key domains: randomization process, deviations from intended interventions, missing outcome data, measurement of outcomes, and selection of reported results. Each domain was judged as having low risk (−), some concerns (+), or high risk of bias (X). The overall risk of bias for each study was then determined according to the results across all domains.

Two reviewers independently (J.V.S.R. and D.S.B.) performed the assessments, and disagreements were resolved by consensus with a third reviewer (R.S.M.). The results of the risk-of-bias evaluation were graphically summarized using Review Manager (RevMan) version 5.4 (https://training.cochrane.org/online-learning/core-software-cochrane-reviews/revman/revman-5-download (accessed on 1 December 2025)) to provide a clear overview of the methodological quality of all included studies.

### 2.7. Quantitative Synthesis and Statistical Analysis

When the data from individual studies were sufficiently homogeneous regarding study design, intervention, and outcome measurement, a quantitative synthesis was performed using Review Manager (RevMan) version 5.4. Continuous outcomes, such as PPD reduction and CAL gain, were expressed as mean differences or standardized mean differences with corresponding 95% confidence intervals.

The level of heterogeneity among the included studies was assessed using the chi-square test and quantified with the I^2^ statistic. An I^2^ value below 25% was considered indicative of low heterogeneity, values between 25% and 50% reflected moderate heterogeneity, and values above 50% indicated substantial heterogeneity. In the presence of low heterogeneity, a fixed-effect model was used, whereas a random-effects model was applied when heterogeneity was moderate or high.

Planned exploratory subgroup analyses were considered according to compound class, follow-up duration, and disease severity, and sensitivity analyses using leave-one-out approaches were initially envisaged. However, due to substantial heterogeneity in intervention types, outcome definitions, and limited numbers of studies within individual subgroups, these analyses were not performed to avoid statistically underpowered or potentially misleading comparisons.

To reduce clinical heterogeneity and improve interpretability, interventions were stratified according to route of administration: (i) locally delivered adjunctive therapies (e.g., gels, chips, subgingival carriers), (ii) systemic supplementation, and (iii) rinse-based or topical home-use formulations. Quantitative pooling was restricted to studies within the same administration category that demonstrated sufficient clinical and methodological comparability, including similar follow-up intervals and outcome reporting. When heterogeneity in delivery modality or study design precluded meaningful aggregation, results were synthesized narratively rather than meta-analyzed.

Several included trials employed split-mouth or split-pocket designs, in which multiple sites within the same patient were allocated to different interventions. These designs introduce within-subject correlation that differs from parallel-group randomized trials. When sufficient summary statistics were available, data were extracted as reported by the study authors. Because most split-design trials did not provide the information required to adjust for intra-patient correlation, effect estimates were pooled using reported values while acknowledging this limitation. The potential influence of non-independence on precision estimates was considered when interpreting pooled outcomes.

For trials with multiple intervention arms sharing a single control group, care was taken to prevent double-counting of participants in the meta-analysis. Control groups were not entered multiple times without adjustment. When more than one comparison from the same study was included, the shared control group was proportionally divided across comparisons, or intervention arms were combined when clinically appropriate, in accordance with Cochrane recommendations. This approach ensured statistical independence and avoided artificial inflation of precision estimates.

### 2.8. Assessment of Certainty of the Evidence

The certainty of evidence for each primary outcome was evaluated using the Grading of Recommendations, Assessment, Development, and Evaluation (GRADE) framework [88]. This approach considers five domains: risk of bias, inconsistency, indirectness, imprecision, and publication bias. Each outcome was classified as high, moderate, low, or very low certainty, depending on the cumulative assessment across these domains. Evidence was downgraded when substantial methodological limitations or inconsistencies were present, or when confidence intervals were wide.

A Summary of Findings (SoF) table was developed using GRADEpro GDT software, version 2023 (https://gradepro.org) to provide a transparent and comprehensive presentation of the overall certainty and strength of evidence supporting each outcome measure.

## 3. Results

Overall, the quantitative synthesis demonstrated that adjunctive use of selected natural products in combination with SRP resulted in statistically significant, albeit generally modest, improvements in key periodontal clinical parameters. Meta-analyses indicated consistent short-term benefits for PPD reduction and CAL gain with agents such as curcumin-based formulations, omega-3 fatty acids, resveratrol, *Aloe vera*, and selected antioxidant-rich supplements, whereas evidence for other herbal preparations was more variable or limited. Improvements were most consistently observed at short- to medium-term follow-up intervals, with fewer studies reporting sustained long-term effects.

### 3.1. Selection of Studies

A total of 8253 records were retrieved from MEDLINE/PubMed, Scopus, Web of Science, Cochrane CENTRAL, Embase, Scielo, and Google Scholar. After removing 2137 duplicates, 6116 unique studies remained for title screening. Of these, 5962 were excluded because they did not satisfy the predefined eligibility criteria. The remaining 154 articles proceeded to abstract evaluation, from which a subset was selected for full-text assessment. Twenty-nine studies were subsequently excluded. Of the remaining 125 articles assessed in full, 35 were further excluded for reasons detailed in Figure 1. In the end, 90 studies met the inclusion criteria and were included in the qualitative synthesis. Of these, 33 studies meet the inclusion criteria for meta-analysis to evaluate quantitative synthesis of clinical parameters.

### 3.2. General Characteristics of the Included Studies

A total of ninety clinical studies published between 2008 and 2025 were included, encompassing randomized controlled trials, split-mouth and split-pocket studies, placebo-controlled trials, and pilot trials (Table 2A–C). The majority followed a randomized controlled design, with parallel-group trials most common, while split-mouth designs were used when local drug-delivery systems were evaluated. Sample sizes ranged widely, from small exploratory cohorts with fewer than 20 participants to larger controlled studies involving more than 100 individuals, reflecting substantial heterogeneity in methodological rigor and statistical power. The included trials enrolled adult patients with chronic periodontitis, Stage II–IV periodontitis, generalized periodontitis, gingivitis with periodontal involvement, or persistent periodontal pockets after initial therapy. The variation in periodontitis diagnoses across the included trials reflects the evolution of case definitions over time. Earlier studies commonly used terms such as chronic or generalized periodontitis, or described conditions such as periodontal involvement or persistent periodontal pockets after initial therapy. In contrast, the current standardized classification introduced by the EFP and the American Academy of Periodontology in 2018 defines periodontitis using a staging and grading system [20]. Because many of the included studies were conducted before the adoption of this contemporary framework, differences in diagnostic terminology and criteria were expected. Several studies specifically targeted medically compromised populations, including individuals with type 2 diabetes mellitus [70,72,89,90,91,92], smokers [61,93,94], rheumatoid arthritis [8], and individuals with Down syndrome [95], while others included systemically healthy participants as controls. Collectively, the studies represented a broad global distribution, with research conducted across India, Egypt, Iran, Thailand, Brazil, Serbia, Italy, Germany, and China, reflecting sustained international interest in natural adjunctive therapies for periodontal treatment.

A wide range of natural compounds and biologically active preparations were investigated, spanning herbal extracts, plant-derived bioactives, nutraceutical supplements, essential oils, phenolics, and multi-herbal formulations. Curcumin remained the most extensively studied compound and was delivered in diverse systems, including gels, nanoparticles, nanofibers, strips, chips, collagen carriers, and as a photosensitizer in photodynamic therapy [58,96,97,98,99,100,101,102,103,104]. Numerous trials also evaluated other topical botanical agents, including *Aloe vera*, propolis in gel, extract, nanoparticle, or mouthwash form, green tea catechins, matcha, pomegranate derivatives, mangosteen (*Garcinia mangostana*) gel, *Ocimum sanctum* (Tulsi) extracts, psidium guajava, grape seed extract, spirulina, tea tree oil, chicory extract, miswak chips, and frankincense-based formulations. These interventions covered nearly the entire range of included studies and collectively demonstrated adjunctive benefits, including reduced clinical inflammation, enhanced plaque control, and improved periodontal pocket metrics. Propolis-containing formulations, in particular, showed consistent anti-inflammatory and antimicrobial effects in both systemically healthy and medically compromised populations [70,97,100,101,105,106,107].

Across the included trials, clinical outcomes were consistently assessed using standard periodontal parameters, primarily PPD, CAL, BOP, and gingival inflammation indices. Nearly all studies demonstrated improvements following SRP, with adjunctive natural therapies often providing additional benefits. When stratified by route of administration, distinct patterns emerged in the magnitude and consistency of treatment effects, as demonstrated in Table 2A–C. Overall, adjunctive benefits were most frequently observed for short-term reductions in PPD and inflammatory indices, whereas CAL gains were present but more variable. Microbiological and biochemical outcomes were reported less uniformly and displayed greater heterogeneity, limiting quantitative synthesis. Treatment effects across delivery modalities were predominantly observed within short- to medium-term follow-up intervals (1–6 months), with fewer studies evaluating sustained long-term outcomes [56,59,60,72,75,77,81,82,83,84,85,86,87,88,89,93,96,103,104,105,106,107,108,109,110,111,112,113,114,115,116].

**Table 2:** (**A**). Locally delivered summary of the included studies in this systematic review. (**B**). Systemic supplementation summary of the included studies in this systematic review. (**C**). Rinse/topical adjuncts summary of the included studies in this systematic review.

**(A). Locally delivered summary of the included studies in this systematic review**							
**Author/Year**	**Study Design**	**Population/Study Location**	**Study Groups**	**Intervention/Exposure**	**Methods**	**Main Findings**	**Conclusion**
Thippeswamy et al., 2025 [102]	Parallel-group RCT	20 patients (40 sites) with chronic periodontitis; India	1. Test: SRP + 8% *O. sanctum* gel 2. Control: SRP alone	Adjunctive local drug delivery of 8% *Ocimum sanctum* (Tulsi) gel.	Clinical parameters (PPD, RAL, PI, GI, GBI) and microbiological colony counts (*A. a.*, *P. g.*) assessed at baseline, 1, and 3 months.	- Clinical: Test group showed significantly greater reduction in PPD (6.40 ± 0.52 to 2.6 ± 0.48 mm) and gain in RAL (8.4 ± 0.52 to 4.6 ± 0.48 mm) - Microbiological: Greater reduction in bacterial counts in test group, but intergroup difference was not significant.	8% *O. sanctum* gel is an effective clinical adjunct to SRP, significantly improving clinical parameters, though microbiological superiority was not statistically significant.
Dubey et al., 2025 [117]	Split-pocket RCT	31 patients (75 sites) with chronic periodontitis; India	1. Group I: SRP + Blank nanofiber (Placebo) 2. Group II: SRP + Ashvakatri-loaded nanofiber (Test) 3. Group III: SRP + Tetracycline-loaded nanofiber (Standard)	Adjunctive placement of a sustained-release nanofiber scaffold loaded with either an herbal extract (Ashvakatri) or tetracycline.	Clinical parameters (PI, GI, PPD, CAL) assessed at baseline, 1, and 2 months.	- Group II and Group III showed significant improvements in PPD and CAL compared to Group I at 1 and 2 months (*p* < 0.05). - No significant difference was found between the Test and Standard groups.	The Ashvakatri-loaded nanofiber scaffold is as effective as the tetracycline-loaded scaffold and superior to placebo, making it a promising and cost-effective alternative for LDD in periodontitis.
Gupta et al., 2025 [93]	RCT	40 smoker patients with chronic periodontitis; India	1. Test: SRP + 2% Curcumin gel 2. Control: SRP alone	SRP performed for all. Test group received subgingival curcumin gel application at baseline and 4 weeks in pockets ≥ 5 mm.	Clinical parameters (PPD, CAL, GI) assessed at baseline, 4 weeks, and 8 weeks.	- The SRP + Curcumin group showed significantly greater reductions in PPD and GI, and greater improvement in CAL at 4 and 8 weeks compared to SRP alone.	Subgingival delivery of 2% curcumin gel is a highly effective adjunct to SRP for improving periodontal health in smokers.
Petrović et al., 2025 [103]	Randomized prospective study RCT	60 patients with Stage II, Grade A periodontitis; Serbia	1. Intervention (n = 30): SRP + Herbal Tincture 2. Control (n = 30): SRP alone	Tinctura paradentoica^®^ (a blend of 7 herbal tinctures & peppermint oil) applied via subgingival irrigation (0.1 mL/pocket) for 5 consecutive days after SRP.	Clinical indices: PI, BOP, and CAL. Microbiological: PCR for *P. g.*, *T. f.*, *T. d.* Chemical: HPLC analysis of tincture.	- Significant reduction in *T. f.* and *T. d.* in intervention group. No change for *P. g*. - Significantly greater reduction in PI and BOP in intervention group at 1 month. CAL improvement was statistically but not clinically significant.	Adjunctive use of the herbal tincture with SRP provides enhanced short-term reduction in PI, BOP, and specific periodontopathogens, showing potential as a supportive therapy.
Scribante et al., 2025 [95]	Randomized, placebo-controlled trial RCT	40 patients with Down Syndrome and gingival inflammation; Italy	1. Trial (n = 20): SRP + Postbiotic Gel 2. Control (n = 20): SRP + Placebo Gel	Daily home application of an intensive soothing gel containing postbiotics, lactoferrin, zinc, and natural compounds vs. a placebo gel without active ingredients for 6 months.	Clinical indices: BOP, Plaque Control Record (PCR), MGI, and Mobility (Miller). Compliance & Satisfaction: VAS.	- Primary Outcome: BOP was significantly lower in the Trial group at the 6-month intergroup evaluation. - No other significant intergroup differences. Both groups showed intragroup improvements.	A postbiotic-based gel is a valuable adjunct to SRP for improving periodontal health (specifically reducing BOP) in patients with Down syndrome over 6 months.
Sanjay et al., 2025 [118]	Split-Mouth RCT	30 patients with chronic periodontitis; India	1. Test Sites: SRP + Curcumin Gel 2. Control Sites: SRP alone	Curcumin gel placed into periodontal pockets after SRP at test sites, sealed with periodontal dressing. Control sites received SRP only. Follow-up at 1 and 3 months.	Clinical indices: PI, GI, PPD. Measurements at baseline, 1 month, and 3 months.	- Significant PI improvement in both groups over time, but no significant difference between groups. - Test sites showed a statistically significant greater improvement in GI and PPD reduction compared to control sites at 1 and 3 months.	Locally delivered curcumin gel is an effective adjunct to SRP, resulting in significant improvements in gingival inflammation and pocket depth reduction over 3 months.
Grassi et al., 2025 [119]	Double-blind, RCT	27 patients (40 periodontal pockets) with stage II–IV periodontitis; Italy	1. Group A: SRP + placebo gel 2. Group B: SRP + AOEOO gel	Adjunctive subgingival application of AOEOO gel vs. placebo, after full-mouth SRP.	Clinical indices (PPD, CAL, PI, BOP) at baseline, 3, 6 months; microbiological sampling (PCR for 6 pathogens) at baseline and 6 months.	AOEOO group showed significant improvements in all clinical indices at 3 and 6 months vs. placebo. Reduction in total bacterial load (40.6%) and all 6 key pathogens (63.8–98.7%).	AOEOO gel is a safe and effective adjunct to SRP, improving periodontal outcomes and reducing pathogenic bacteria.
Pauly et al., 2025 [120]	Split-mouth, RCT	20 patients with chronic periodontitis; India	1. Group 1: SRP + placebo gel (single application) 2. Group 2: SRP + 5% pomegranate gel (single application) 3. Group 3: SRP + 5% pomegranate gel (applications at baseline & 3 months)	Locally delivered 5% pomegranate gel vs. placebo, adjunctive to SRP.	Clinical parameters (PPD, RAL, PI, GI, BOP) measured at baseline, 3, and 6 months.	All groups improved, but Group 3 showed greatest reduction in PPD and RAL at 6 months. Pomegranate gel significantly improved clinical outcomes vs. placebo.	5% pomegranate gel is effective as an adjunct to SRP in chronic periodontitis, with reapplication enhancing results.
Gunjal et al., 2024 [121]	Single-blind, parallel-arm RCT	180 patients (35–55 yrs) with stage II periodontitis; India	1. Group A: SRP + 1% CHX gel 2. Group B: SRP + 16% MA gel Group C: SRP + placebo gel	Full-mouth SRP + gel application at baseline, 15, and 30 days.	Clinical (PI, GI, PPD) and microbiological (*A. a.*, *P. g.*, *T. f.*) assessments at baseline and 45 days.	No significant difference between MA and CHX groups for PI, GI, or microbial load. MA showed greater PPD reduction (mean diff 0.18 mm). Both MA and CHX were superior to SRP alone.	MA gel is as effective as CHX gel as an adjunct to SRP in stage II periodontitis, with comparable anti-inflammatory and antimicrobial effects.
Thakuria et al., 2024 [122]	Split-mouth design, RCT	20 patients (18–65 yrs) with chronic periodontitis; India	1. Group A: SRP + saline 2. Group B: SRP + 100% *Aloe vera* gel 3. Group C: SRP + tetracycline fibers	SRP + local delivery of gel/fibers/saline.	Clinical (PPD, RAL, PI, mSBI) assessments at baseline, 7 days, and 3 months.	*Aloe vera* and tetracycline groups showed significant PPD reduction vs. saline. Tetracycline group had greater PI and mSBI reduction vs. saline. No significant difference between *Aloe vera* and tetracycline groups.	*Aloe vera* gel is as effective as tetracycline fibers as an adjunct to SRP and may be a cost-effective herbal alternative.
Rathod et al., 2024 [123]	Split- mouth, RCT	12 patients (44 intrabony defects) with Stage III periodontitis; India	1. Group I: SRP + placebo gel; 2. Group II: SRP + herbal gel (*Picrorhiza kurroa* + *Ficus bengalensis*)	Herbal gel applied locally after SRP; follow-up at 3 and 6 months.	Clinical (PI, mSBI, PPD, CAL) and radiographic measurement.	Herbal gel group showed significantly greater bone fill, PPD reduction, and CAL gain vs. placebo at 6 months.	Herbal gel is more effective than SRP alone in improving clinical and radiographic outcomes in Stage III periodontitis.
Al-Bayaty et al., 2024 [124]	Single-blind RCT	12 male patients (240 periodontal pockets); Malaysia	1. Control (n = 60): SRP alone 2. Chitosan (n = 60): SRP + plain chitosan chip 3. Salvadora persica (n = 60): SRP + Salvadora persica chip 4. BITC (n = 60): SRP + Benzyl Isothiocyanate chip	Biodegradable chips were inserted into periodontal pockets (≥5 mm) twice (at baseline and day 14) as an adjunct to SRP.	PI, BOP, PPD, and CAL were measured at baseline and day 60.	- All groups showed significant improvements. - The Salvadora persica chip group showed the greatest reduction in PPD (1.55 mm) and the highest gain in CAL (1.55 mm) compared to other groups.	Salvadora persica-based biodegradable chips are an effective adjunct to SRP, showing superior results in reducing pocket depth and improving CAL compared to SRP alone or with other chips.
Agrawal et al., 2024 [125]	Split-mouth RCT	30 patients (90 sites); India	1. Category 1 (n = 30): SRP alone 2. Category 2 (n = 30): SRP + Curcumin gel 3. Category 3 (n = 30): SRP + Tulsi extract gel	Locally administered medication (gel) was placed in the periodontal pocket (5–8 mm) after SRP and covered with periodontal pack.	PPD, CAL, PI, GI, mSBI, and microbiological (BAPNA assay for red-complex bacteria) parameters were assessed at baseline and 30 days.	- All treatments significantly improved all parameters. - Tulsi extract resulted in a significantly greater reduction in BAPNA (indicating better action against red-complex bacteria) than SRP alone. - Curcumin gel showed a slightly greater, but not significant, reduction in PI and GI.	Both Tulsi and Curcumin are effective adjuncts to SRP. Tulsi was more effective in reducing pathogenic bacteria, while Curcumin showed a strong anti-plaque effect. Overall, they have comparable efficacy in improving clinical periodontal markers.
Katariya et al., 2024 [126]	Prospective RCT	60 patients with Generalized Chronic Periodontitis; India.	1. Group 1: SRP alone (Control, n = 30) 2. Group 2: SRP + *Aloe vera* Hydrogel (Test, n = 30)	Local Drug Delivery: A single application of *Aloe vera* Hydrogel microbeads into periodontal pockets after SRP.	Clinical: PI, PPD, and CAL. Timing: Baseline and 3 months.	- Both Groups: Significant improvement in PI, PPD, CAL at 3 months vs. baseline. - Intergroup (Test vs. Control): Test group showed significantly better improvement in PPD and CAL at 3 months. No significant difference in PI.	*Aloe vera* hydrogel as an adjunct to SRP is more effective than SRP alone in improving clinical parameters (PPD and CAL) in patients with chronic periodontitis.
Yousef et al., 2024 [104]	Randomized clinical-controlled RCT	20 sites in 10 patients with Moderate Chronic Periodontitis; Egypt.	1. Group I: SRP only (Control, n = 10 sites) 2. Group II: SRP + Frankincense Extract Gel (Test, n = 10 sites)	Local Drug Delivery: Subgingival application of 4% *w*/*v* Frankincense Extract Gel after SRP on day 1, 7, and 14.	Clinical: PPD, CAL, BOP. Microbiological: Load of *P. g.* in GCF via PCR. Timing: Baseline, 1 month, and 3 months.	- Group II: Significantly greater reduction in PPD and gain in CAL at 1 and 3 months vs. control. Significantly greater reduction in BOP at 3 months. - Microbiological: Significant reduction in *P. g.* load at 1 and 3 months in Group II, but not in the control group.	Subgingival application of Frankincense extract gel is an effective adjunct to SRP, providing significant clinical and microbiological (anti-*P. g.*) improvements in moderate chronic periodontitis.
Aggarwal et al., 2023 [105]	Randomized controlled clinical trial RCT	30 CP patients with PPD ≥ 5 mm; India	1. Group 1: SRP only 2. Group 2: SRP + 940 nm Diode Laser 3. Group 3: SRP + Propolis Gel	Laser: 1.5 W, 940 nm, 30 sec/pocket Propolis: Ethanolic extract formulated as a gel, delivered subgingivally.	Clinical parameters (PI, GI, PPD, CAL) assessed at baseline, 1, and 3 months.	- All groups showed significant improvements. SRP + Laser showed the best results in reducing gingival inflammation, PPD, and CAL - Propolis gel also showed encouraging clinical results but was less effective than laser.	Lasers as an adjunct to SRP were highly effective. Propolis gel is a promising natural alternative but requires more long-term studies.
Sahu et al., 2023 [106]	Double-Blind RCT	40 patients with periodontitis (Stage II/III) and PPD 4–6 mm; India	1. Test Group: SRP + Propolis Nanoparticles (PRO) 2. Control Group: SRP + Saline (Placebo)	Subgingival delivery of propolis nanoparticles (size: 88–103 nm) in liquid solution. Pocket sealed with cyanoacrylate.	Clinical parameters (PI, GI, BOP, PPD, RAL) assessed at baseline, 1, and 3 months.	- The SRP + PRO group showed significantly greater improvement in GI, BOP, PPD, and RAL at 3 months compared to SRP + Placebo. - PI showed improvement at 3 months.	Subgingival delivery of propolis nanoparticles is a highly effective adjunct to SRP, resulting in significant clinical improvements in periodontitis patients.
Abdel-Fatah et al., 2023 [57]	Randomized controlled clinical trial RCT	54 patients (36 with Stage II Grade A periodontitis, 18 healthy); Egypt	1. Group I: Healthy (Negative Control) 2. Group II: SRP only 3. Group III: SRP + 2% Curcumin Gel	Subgingival delivery of 2% curcumin gel weekly for 4 weeks after full-mouth SRP.	Clinical parameters (PI, GI, PPD, CAL) and salivary Procalcitonin (PCT) levels assessed at baseline and 6 weeks.	- Group III showed significantly greater improvement in PI, PPD, and CAL than Group II - Salivary PCT levels decreased significantly in both periodontitis groups, with no significant difference.	Curcumin gel as an adjunct to SRP is effective and significantly improves clinical parameters. Salivary PCT is a useful inflammatory biomarker for periodontitis.
Borgohain et al., 2023 [127]	Split-mouth, RCT	10 systemically healthy patients with chronic periodontitis; India	Three sites/patient: 1. Group I: SRP alone 2. Group II: SRP + Curcumin gel LDD 3. Group III: SRP + 99% *Aloe vera* gel LDD	Single application of curcumin or *Aloe vera* gel into periodontal pockets after SRP.	Clinical parameters (PPD, CAL, PI, GI, mSBI) were recorded at baseline and 30 days.	All groups improved. *Aloe vera* gel (Group III) showed the most statistically significant improvement in PPD, GI, and mSBI compared to SRP alone. Curcumin also showed benefits.	Both curcumin and *Aloe vera* gel as LDD adjuncts to SRP are effective, with 99% *Aloe vera* extract producing particularly significant clinical improvements.
Elsadek et al., 2023 [96]	Double-blinded, RCT	75 patients with Stage III Grade B periodontitis; Saudi Arabia	1. Group I: SRP + PGA/MB/AV 2. Group II: SRP + Antimicrobial PDT 3. Group III: SRP alone	Group I received subgingival delivery of methylene blue-loaded nanoparticles in *Aloe vera* gel. Group II received PDT with methylene blue.	Clinical parameters (PI, BOP, PPD, CAL) and levels of *T. f.* and *P. g.* were measured at baseline, 3, and 6 months.	Group I PGA/MB/AV showed the most significant PPD reduction and CAL gain, and a significant reduction in *T. f*. Group II PDT showed the best improvement in BOP.	Methylene blue-loaded nanoparticles in *Aloe vera* gel as an adjunct to SRP produced superior periodontal healing and microbial reduction compared to PDT or SRP alone in severe periodontitis.
Manjunatha et al., 2022 [128]	Double-blinded, placebo-controlled, split-mouth, RCT	25 patients with Stage II Grade B periodontitis; India	1. Experimental: SRP + 4% mangosteen gel (LDD) 2. Control: SRP + placebo gel (LDD)	Single subgingival application of 4% mangosteen gel or placebo after full-mouth SRP.	Clinical parameters (PI, GBI, PPD, RAL) and GCF total antioxidant capacity (TAC) were measured at baseline and 3 months.	The experimental group showed significantly greater reduction in PPD and RAL and a significant increase in GCF TAC (from negative to positive values) compared to control. Both groups improved in PI and GBI.	Adjunctive 4% mangosteen gel LDD significantly improves clinical parameters and antioxidant capacity in GCF, supporting its use in managing moderate chronic periodontitis.
Verma et al., 2022 [129]	Split-mouth, RCT	30 patients with generalized chronic periodontitis (90 sites); India	Group 1: SRP + *Acmella oleracea* gel (1%); Group 2: SRP + *Acaia catechu* gel (1%); Group 3: SRP only (control)	Local drug delivery of herbal gels after SRP; single application at baseline.	Clinical (GI, PI, GBI, PPD, CAL, RAL) at baseline, 1, 3, 6 months; microbiological (BANA test) at baseline and 6 months.	All groups improved clinically; Group 2 (*Acacia catechu*) showed better PPD and CAL reduction. Microbiological reduction was nonsignificant.	Both herbal gels improve clinical parameters when combined with SRP; Acacia catechu performed slightly better.
Kaipa et al., 2022 [130]	Parallel trial, RCT	60 patients with chronic periodontitis; India	Group I: SRP + placebo (SRP-P); Group II: SRP + spirulina microspheres (SRP-S)	Subgingival placement of spirulina microspheres (2 mg) after SRP.	Clinical (GI, BOP, PPD, CAL) and biochemical (salivary and serum MDA) at baseline and 90 days.	SRP-S group showed significantly greater improvement in clinical parameters and salivary MDA vs. SRP-P. Serum MDA reduced in both groups.	Spirulina local delivery adjunctive to SRP has potent antioxidant effects and improves periodontal health.
Saini et al., 2021 [131]	Split-mouth RCT	30 patients (90 sites); India	1. Group I: SRP + Neem Chip 2. Group II: SRP + Turmeric Chip 3. Group III: SRP + Placebo Chip	Indigenous biodegradable chips (4 × 2 mm) containing 5% neem or turmeric extract.	Clinical parameters (PI, GI, PPD, RAL) recorded at baseline, 1 month, and 3 months.	- At 1 month, Groups I & II showed significant improvement in PI, GI, PPD, and RAL compared to Group III. - At 3 months, the intergroup difference was not significant, though improvements were maintained.	Neem and turmeric chips as an adjunct to SRP are more effective than SRP alone in the short term (1 month). Long-term benefits are comparable to SRP alone.
Das et al., 2021 [132]	Parallel-group RCT	72 patients (72 sites); India & Italy	1. Test Group: SRP + 4% GSE solution 2. Control Group: SRP alone	Intra-pocket delivery of 4% GSE solution, sealed with cyanoacrylate.	Clinical parameters (PI, GI, PPD, RAL) recorded at baseline and 3 months.	- Test group showed statistically significant greater reduction in PPD and gain in RAL at 3 months compared to control. - No significant difference in PI and GI.	A single intra-pocket application of 4% GSE as an adjunct to SRP is beneficial in reducing probing depth and improving attachment levels in periodontal pockets.
Sharma et al., 2021 [114]	Split-mouth RCT	15 patients (30 sites); India	1. Test Site: SRP + 3% Psidium guajava Gel 2. Control Site: SRP alone	Local delivery of indigenously prepared 3% Psidium guajava in situ gel.	Clinical parameters (PI, GI, BOP, PPD, CAL) and microbiological counts (*A. a.*, *P. g.*) assessed at baseline, 1, and 3 months.	- Test sites showed significantly greater reduction in PPD, BOP, and gain in CAL at 3 months. - Microbiological analysis showed a significantly greater reduction in *A. a.* and *P. g.* counts in test sites.	Adjunctive use of 3% Psidium guajava gel is effective in improving clinical and microbiological parameters in the management of chronic periodontitis.
George et al., 2021 [133]	Parallel-group RCT	15 patients per group; India	1. Test Group: SRP + *Ocimum sanctum* Nanofibers 2. Control Group: SRP alone	Local application of electrospun *Ocimum sanctum* (10% wt) nanofibers in persistent pockets.	Clinical parameters (GI, BOP, PPD, CAL) and GCF IL-1β levels assessed at baseline and 1 month.	- No significant intergroup difference in GI, BOP, PPD, or IL-1β levels. However, the test group showed a statistically significant greater gain in CAL compared to the control.	*Ocimum sanctum* nanofibers provide an additional benefit in CAL gain when used as an adjuvant to SRP, suggesting a positive anti-inflammatory effect.
Pérez-Pacheco et al., 2021 [58]	Split-mouth, Double-blind, Placebo-controlled RCT	20 patients (80 sites); Brazil	1. Test Group (N-Curc): SRP + Curcumin-loaded Nanoparticles (50 µg) 2. Control Group: SRP + Empty Nanoparticles	Single local application of 100 µL of nanoencapsulated curcumin or placebo (empty nanoparticles).	Clinical parameters (PPD, CAL, BOP), GCF cytokines (IL-1α, IL-6, TNF-α, IL-10), and subgingival microbiota (40 species) monitored over 180 days.	- Both groups showed significant clinical improvement with no significant differences between them. - N-Curc group showed a trend for lower IL-6 and TNF-α, a specific increase in *V. p.*, and prevented *A. a.* regrowth.	A single application of nanoencapsulated curcumin provided no significant additive clinical benefit to SRP in systemically healthy, non-smoking patients, despite some positive immunologic and microbiological trends.
Taalab et al., 2021 [134]	Parallel randomized controlled clinical trial RCT	30 patients; - Test: 15 - Control: 15; Egypt	1. SRP alone 2. SRP + 5% Tea Tree Oil Gel	Single intrapocket application of 5% Tea Tree Oil gel after SRP.	Clinical: PPD, CAL, GI, BOP. Biochemical: GCF levels of MMP-8 (ELISA). Timing: Baseline, 1, 3, and 6 months.	- Both groups improved. Test group showed significantly greater reduction in CAL at 6 months, and GI & BOP at 3 and 6 months. - GCF MMP-8 levels were significantly lower in the test group at 6 months.	A single application of Tea Tree Oil gel adjunctive to SRP is safe and effective, providing better and sustained clinical and biochemical improvement for up to 6 months.
Rahalkar et al., 2021 [135]	Split-mouth RCT	15 patients; - 45 sites total; India	1. SRP alone (Group 1) 2. SRP + Curcumin Gel (Group 2) 3. SRP + Tulsi Gel (Group 3)	LDD of Curenext^®^ gel (Curcumin) or 10% Tulsi (*Ocimum sanctum*) extract gel after SRP.	Clinical: PPD, CAL, PI, GI, mSBI. Microbiological: BAPNA assay (for red complex bacteria). Timing: Baseline and 30 days.	- All groups improved. Curcumin group had significantly better PI reduction vs. SRP alone. Tulsi group had significantly better mSBI and BAPNA reduction vs. SRP alone. - Tulsi showed better antimicrobial effect; Curcumin showed better anti-plaque effect.	Both Curcumin and Tulsi are effective LDD agents. Curcumin excels in plaque control, while Tulsi is superior in reducing bleeding and red complex bacteria.
Tyagi et al., 2021 [136]	Prospective RCT	30 patients; - Group 1: 10 - Group 2: 10 - Group 3: 10 India	1. SRP + Pomegranate Chip 2. SRP + Pomegranate Gel 3. SRP alone (Control)	LDD of a biodegradable chip or a 10% gel, both containing pomegranate (Punica granatum) extract.	Clinical: PI, GI, PPD, and RAL. Timing: Baseline, 21 days, and 45 days.	- All groups improved. The chip group showed significant early PI reduction (21 days). Chip and gel groups showed significant improvement in RAL. - The chip was most effective in improving RAL.	Pomegranate chip and gel are effective adjuncts. The chip provided superior results, especially in improving CALs, compared to the gel and SRP alone.
Qamar et al., 2021 [137]	RCT	150 patients; Saudi Arabia	1. SRP alone 2. SRP + PDT 3. SRP + *Aloe vera* Gel	PDT: Indocyanine Green photosensitizer + 810 nm diode laser. *Aloe vera*: LDD of fresh *Aloe vera* gel into pockets.	Clinical: PI, BOP, PPD, CAL. Biochemical: GCF levels of IL-6, IL-8, TNF-α (ELISA). Timing: Baseline, 3 months, 6 months.	- Groups 2 and 3 showed significant reduction in inflammatory cytokines (IL-6, IL-8, TNF-α) vs. Group 1. - PDT was most effective in reducing PPD and improving CAL. *Aloe vera* gel was most effective in reducing BOP.	Both PDT and *Aloe vera* gel are effective adjuncts. PDT is better for pocket reduction and gain of attachment, while *Aloe vera* gel is highly effective for reducing gingival inflammation and BOP.
Guru et al., 2020 [138]	Pilot RCT	45 patients; India	1. Group 1: SRP only (n = 15) 2. Group 2: SRP + 1% CHX gel (n = 15) 3. Group 3: SRP + 2% CUR nanogel (n = 15)	Single subgingival application of the respective gel after full-mouth SRP.	Clinical parameters (PI, GI, PPD, CAL) and microbiological analysis (multiplex PCR for *A. a.*, *P. g.*, *T. f.*) at baseline, 21, and 45 days.	- Both LDD groups (CHX & CUR) showed significant improvement in all clinical and microbiological parameters vs. SRP alone. - No significant difference in clinical outcomes between CHX and CUR groups. - Both LDD agents showed comparable antibacterial effects.	2% CUR with a nanocarrier is as effective as 1% chlorhexidine gel as an adjunct to SRP, making it a promising natural LDD agent.
Nakao et al., 2020 [97]	Double-blind, Controlled RCT	23 patients; Japan	1. Group 1: Placebo ointment (n = 6) 2. Group 2: Propolis ointment (n = 6) 3. Group 3: Curry leaf ointment (n = 5) 4. Group 4: Minocycline ointment (n = 6)	Subgingival administration of ointments 3 times at one-month intervals during supportive therapy.	Microbiological analysis (PCR for total bacteria and 6 pathogens in GCF) and clinical parameters (PPD, CAL, etc.) at baseline and 3 months.	- Propolis group: Significant improvement in PPD and CAL; 3/6 *P. g*. positive patients became negative. - Minocycline group: Significant PPD improvement, but not CAL. - Curry leaf group: No significant clinical improvement.	Topical propolis significantly improved clinical parameters and reduced *P. g.*, suggesting it is an effective alternative adjunctive treatment.
Aljuboori & Mahmood, 2020 [139]	Split-mouth RCT	14 patients (28 sites); Iraq	1. Control Site: SRP only 2. Test Site: SRP + 2% Salvia officinalis (Sage) gel	Subgingival application of gel at baseline and 1 week.	Clinical parameters (PI, GI) and immunological analysis (GCF TGF-β1 levels via ELISA) at baseline, 1 week, and 1 month.	- Test group showed significant reduction in GI at 1 week and 1 month. - No significant change in PI in either group. - Test group showed a significant reduction in TGF-β1 levels at 1 month.	Salvia officinalis gel has anti-inflammatory effects in chronic periodontitis, as evidenced by improved gingival health and reduced TGF-β1.
Mohammad, 2020 [140]	RCT	60 patients + 30 healthy controls; Iraq	1. Group A (Test): SRP + 2% CUR gel (n = 30) 2. Group B (Control): SRP only (n = 30) 3. Group C: Healthy controls (n = 30)	Subgingival application of CUR gel after SRP, covered with Coe-pack for 7 days.	Clinical parameters (PI, GI, BOP, PPD, CAL) and serum analysis (Zinc, Magnesium, Copper, IL-1β, TNF-α) at baseline and 1 month.	- Chronic periodontitis patients had altered micronutrients and elevated cytokines vs. healthy controls. - Group A showed significantly greater improvement in all clinical parameters, reduction in Copper, IL-1β, TNF-α, and increase in Zinc and Magnesium than Group B.	CUR gel as an adjunct to SRP is effective in improving clinical parameters, reducing pro-inflammatory cytokines, and restoring balance of essential micronutrients.
Farhood et al., 2020 [141]	Split-mouth RCT	20 patients (9 male, 11 feminine) with periodontitis. Iraq	1. Test: SRP + CUR Oral Gel 2. Control: SRP alone	Subgingival application of CUR oral gel (Curenext^®^) twice, one week apart, as an adjunct to SRP.	Clinical parameters (PI, GI, BOP, PPD, RAL) measured at baseline and 1 month.	- Intra-group: Significant improvement in all clinical parameters (PI, GI, PPD, RAL) in both groups at 1 month. - Inter-group: No significant difference in PI, GI, PPD, RAL between groups. - BOP: Significant reduction only in the test group.	CUR gel is a promising adjunct to SRP, showing a potent effect on clinical parameters, with a significant specific benefit in reducing BOP.
Niazi et al., 2020 [142]	Parallel group, randomized controlled clinical trial RCT	73 patients with chronic periodontitis; Saudi Arabia	Group I (PDT + SRP), Group II (Salvadora Persica gel + SRP), Group III (SRP alone)	PDT with indocyanine green or Salvadora Persica gel applied in pockets, adjunct to SRP.	Clinical (PI, BOP, PPD, CAL) and biochemical (IL-6, TNF-α) parameters at baseline, 3 and 6 months.	Group I (PDT) showed significant PPD reduction and CAL gain. Group II (Salvadora Persica) showed significant BOP reduction. Both reduced IL-6 and TNF-α vs. control.	Both PDT and Salvadora Persica gel reduce periodontal inflammation; PDT improves CAL, while SPR reduces BOP.
Elsadek et al., 2020 [98]	Single-blind, parallel, RCT	87 patients with periodontitis; Saudi Arabia and Egypt	1: PDT + SRP; 2: *Aloe vera* gel + SRP; 3: SRP only	PDT (3 sessions with methylene blue + diode laser) vs. topical *Aloe vera* gel vs. SRP alone.	Clinical (PI, BOP, PPD, CAL) and microbiological (*T. f.*, *P. g.*) assessments at baseline, 3 months, 6 months.	PDT significantly improved PPD and CAL vs. other groups; *Aloe vera* significantly reduced BOP; PDT reduced pathogens at 3 months; *Aloe vera* reduced *T. f.* (3 months).	Both adjuncts reduced inflammation; *Aloe vera* improved bleeding; PDT improved attachment and pathogen reduction.
Mahendra et al., 2020 [115]	Double-masked, split-mouth, RCT	26 patients with chronic periodontitis; India	Split-mouth: Test site (SRP + 3% Ginkgo biloba gel) vs. Placebo site (SRP + placebo gel)	3% Ginkgo biloba gel applied subgingivally after SRP.	Clinical (PPD, CAL, BOP, PI) and microbiological (Herper simplex, Epstein–Barr Virus, Cytomegalo Virus, *P. g.* via PCR) at baseline and 3 months.	Significant reduction in PPD, CAL, BOP, PI and viral/bacterial load in test sites vs. placebo.	Ginkgo biloba gel as local drug delivery improves periodontal status and reduces viral/bacterial pathogens.
Tawfik et al., 2019 [143]	Examiner-masked, RCT	16 chronic periodontitis patients; Egypt	1: SRP + Lycopene SLMs gel; 2: SRP only	Lycopene-loaded SLMs gel applied locally into pockets after SRP.	GCF protein carbonyl (oxidative stress marker) and lycopene concentration via HPLC; clinical parameters (PI, MGI, PPD, CAL, IBD) at baseline, 1 to 6 months.	Lycopene SLMs reduced GCF protein carbonyl levels significantly vs. SRP alone; improved PPD and CAL at 6 months; sustained drug release up to 30 days.	Lycopene SLMs as local antioxidant therapy reduces oxidative stress and improves periodontal health.
Ivanaga et al., 2019 [116]	Split-mouth RCT	25 type 2 diabetic patients with residual pockets; Brazil	1. SRP only 2. SRP + CUR irrigation (CUR) 3. SRP + LED irradiation (LED) 4. SRP + PDT (CUR + LED)	Subgingival irrigation with CUR solution (100 mg/L) and/or LED irradiation (465–485 nm) as an adjunct to a single session of SRP.	Clinical parameters (PPD, CAL, BOP, PI) assessed at baseline, 3, and 6 months.	- Inter-group: No significant differences between the four treatment groups at any time point. - Intra-group: Significant PPD reduction and BOP in all groups at 3 and 6 months. - Significant CAL gain only in the PDT and LED groups at the 3-month.	For diabetic patients, a single session of PDT (CUR + LED) or LED alone as an adjunct to SRP may provide short-term (3-month) benefits in CAL gain.
Raghava et al., 2019 [59]	Split-mouth RCT	10 patients (5M, 5F) with chronic periodontitis; India	1. Test: SRP + CUR Gel 2. Control: SRP alone	Local subgingival delivery of 2% curcumin gel into the pocket post-SRP, covered with periodontal pack.	PI, GI, PPD, and CAL measured at baseline and 4 weeks.	- Statistically significant reduction in PI and PPD in the test group compared to the control. - CAL improved in the test group, but the result was not statistically significant. - GI decreased in both groups.	CUR gel as a local drug delivery adjunct to SRP showed significant improvement in plaque control and pocket depth reduction, making it an effective alternative.
Rayyan et al., 2018 [144]	Double-blind, parallel-group RCT	5 systemically healthy patients (86 periodontal sites); Saudi Arabia	1. Control Group: 38 sites (Control gel) 2. GSE Group: 48 sites (2% GSE gel)	Subgingival application of a 2% mucoadhesive GSE gel at baseline and on days 3, 6, and 9.	Clinical parameters (PI, GI, PPD, BOP) measured at baseline, 4 weeks, and 6 months. All patients received SRP one week before baseline.	- PI and GI: Significant reduction in both groups over time. The reduction was significantly greater in the GSE group at 6 months. - PPD: Significant reduction in both groups over time, but no significant difference between groups. - BOP: No significant improvements.	Subgingival application of 2% GSE gel as an adjunct to SRP showed significant improvement in plaque and gingival inflammation but did not provide significant additional benefit in reducing PPD or BOP.
Singh et al., 2018 [145]	Split-mouth RCT	40 patients (120 sites) with chronic periodontitis; India	1. Group A: SRP + CHX chip 2. Group B: SRP + Turmeric chip 3. Group C: SRP only	Subgingival placement of a biodegradable chip containing either 2.5 mg CHX or 5% Turmeric.	PI, GI, PPD, and RAL recorded at baseline, 1, and 3 months.	- CHX and Turmeric chips were equally effective and both superior to SRP alone in reducing PPD and improving RAL over 3 months. - Group C showed deterioration after 1 month.	Both CHX and turmeric chips are effective adjuncts to SRP. Turmeric chip is a promising, cost-effective alternative to CHX.
Andrade et al., 2017 [107]	RCT	16 individuals with chronic periodontitis; Brazil	1. Test Group: SRP + 20% Propolis extract irrigation (65 teeth) 2. Control Group: SRP + Saline irrigation (62 teeth)	Subgingival irrigation with 20% hydroalcoholic propolis extract vs. saline, applied after SRP and at 15 days.	Clinical parameters (PPD, PI, GI, OHI) recorded at baseline, 45, 75, and 90 days.	- Both groups showed significant improvement over time. - Test Group showed significantly greater PPD reduction than Control Group at 45 and 90 days. - No significant between-group differences for PI, GI, and OHI.	Subgingival irrigation with 20% propolis extract as an adjunct to SRP was more effective than saline in reducing probing depth for up to 90 days.
Mahendra et al., 2017 [99]	Double-masked RCT	50 patients with chronic periodontitis (PPD ≥ 5 mm); India	1. Test Group: SRP + 4% Mangostana (MGA) gel (25 pts) 2. Control Group: SRP + Placebo gel (25 pts)	A single subgingival application of 4% *Garcinia mangostana* pericarp gel or placebo gel after SRP.	Clinical parameters (PPD, CAL, BOP, PI) and red complex microorganisms (via PCR) assessed at baseline and 3 months.	- MGA group showed significantly greater reduction in PPD, CAL, BOP, and *T. d.* compared to the placebo group (*p* ≤ 0.05). - *P. g.* and *T. f*. were not detected.	4% Mangostana gel as an adjunct to SRP significantly improved clinical parameters and reduced *T. d.*, offering a new dimension to periodontal therapy.
Pradeep et al., 2016 [72]	Single-center, randomized, longitudinal, triple- masked, interventional study RCT	60 patients with T2DM and chronic periodontitis; India	1. Group 1 (n = 30): SRP + Placebo Gel 2. Group 2 (n = 30): SRP + *Aloe vera* Gel	LDD of *Aloe vera* gel or placebo gel into periodontal pockets after SRP.	Clinical parameters (PI, mSBI, PPD, CAL) recorded at baseline, 3, and 6 months.	- Group 2 showed significantly greater reductions in PI, mSBI, and PPD at 3 months. - Significantly greater CAL gain in Group 2 at all time intervals (baseline to 3 and 6 months).	Locally delivered *Aloe vera* gel is an effective adjunct to SRP, improving clinical parameters in patients with T2DM and chronic periodontitis.
Elavarasu et al., 2016 [146]	Split-mouth RCT	15 patients with chronic periodontitis (bilateral pockets); India	1. Group I (Control): SRP only 2. Group II (Test): SRP + 0.2% CURStrip 3. Healthy Group (n = 5 sites)	LDD of a 0.2% CUR-loaded collagen strip vs. SRP alone.	Clinical parameters (PI, GI, SBI, PPD) and SOD levels in GCF measured at baseline and 21 days.	- Both test and control groups showed significant clinical improvement. - SOD levels increased significantly in both groups post-therapy.	A 0.2% CUR strip is an effective adjunct to SRP, significantly improving the SOD in the subgingival environment.
Rattanasuwan et al., 2016 [147]	RCT	48 subjects with chronic periodontitis (PD 5–10 mm); Thailand	1. Test Group (n = 24): SRP + Green Tea Gel 2. Control Group (n = 24): SRP + Placebo Gel	LDD of a thermosensitive green tea extract gel or placebo gel after SRP. Applied at baseline, 1 week, and 2 weeks.	Clinical parameters (PPD, CAL, GI, BOP, FMPS) recorded at baseline, 1, 3, and 6 months after last application.	- Both groups showed significant improvement in all parameters. - Test group had significantly lower GI at 1 and 3 months and lower BOP at 3 months. - No inter-group difference in PPD, CAL, or FMPS.	Adjunctive green tea gel provides a superior benefit in reducing gingival inflammation and bleeding compared to SRP alone, but not in PPD reduction or CAL gain.
Grover et al. 2016 [148]	Double-blind, placebo-RCT	40 patients with chronic periodontitis; India	Test: 20 patients (SRP + 10% Emblica officinalis gel); Control: 20 (SRP + placebo gel)	Subgingival application of 10% Emblica officinalis sustained-release gel.	Clinical parameters (PPD, CAL, mSBI) at baseline, 2 and 3 months.	Test group showed significantly greater reduction in PPD, mSBI, deep pockets, and greater CAL gain (*p* < 0.05).	Emblica officinalis gel adjunct to SRP is more effective than SRP alone in reducing inflammation and improving periodontal health.
Sreedhar et al., 2015 [149]	Split-mouth RCT	15 patients with chronic periodontitis (60 sites); India	1. Q1: SRP alone 2. Q2: SRP + CUR gel (5 min) 3. Q3: SRP + CUR + PDT (single application) 4. Q4: SRP + CUR + PDT (multiple applications)	Application of CUR gel with/without photoactivation (470 nm blue light). PDT was applied on day 0 (Q3) or days 0, 7, and 21 (Q4).	Clinical parameters (PI, SBI, PPD, CAL) and microbial culture for Aa and BPB at baseline and 3 months.	- All treatments improved clinical parameters. - Curcumin PDT (especially multiple applications in Q4) showed the greatest reduction in SBI, PPD, and microbial counts of *A. a.* and BPB compared to SRP alone or curcumin without light.	Curcumin photodynamic therapy is a valuable adjunct to SRP. Multiple applications of PDT are more beneficial than a single application in reducing clinical and microbiological parameters.
Yaghini et al., 2014 [150]	Double-blind RCT	18 patients (74 sites) with moderate chronic periodontitis; Iran	1. Test: SRP + Herbal Gel 2. Control: SRP + Placebo Gel	Test Gel: Subgingival delivery of a gel containing 20% Quercus brantii (oak) and 1% Coriandrum sativum (coriander) extract. Applied twice (baseline and 1 week).	Clinical parameters (PPD, CAL, PBI, PI) measured at baseline, 1 month, and 3 months.	- Both groups showed statistically significant intra-group improvements in all clinical indices (PPD, CAL, PBI, PI) at 1 and 3 months. - No statistically significant inter-group differences were found for any parameter at any time point.	The herbal gel containing oak and coriander extracts did not provide a significant clinical advantage over SRP alone as an adjunctive treatment for moderate chronic periodontitis.
Cheng et al., 2014 [151]	RCT	60 patients with moderate to severe periodontitis; China	1. Test: SRP + EGB Gel 2. Positive Control: SRP + Periocline 3. Negative Control: SRP + Blank Gel	LDD; Test: Subgingival application of EGB sustained-release gel. Control: Minocycline ointment (Periocline) or blank gel.	Detection rates of 4 periodontal pathogens (*T. d.*, *T. f.*, *P. i.*, *P. g.*) via PCR at baseline, 1 week, 2, and 4 months. Clinical parameters (PI, BOP, PPD, CAL) at baseline, 3, and 6 months.	- EGB significantly reduced detection rates of all 4 pathogens, with effects comparable to Periocline for *T. d.*, *T. f.*, and *P. i.*, but inferior against *P. g.* short-term. - EGB led to significant improvements in PPD and CAL, comparable to Periocline at 3 months but slightly inferior at 6 months.	EGB significantly inhibits major periodontal pathogens and improves clinical parameters (PPD, CAL). It can be used as an effective adjuvant for periodontitis treatment, offering a herbal alternative to antibiotics.
Sanghani et al. 2014 [100]	Double-blind, RCT	20 patients with chronic periodontitis; India	Test: 20 sites (SRP + propolis); Control: 20 sites (SRP alone)	Subgingival delivery of Indian propolis extract.	Clinical (GI, BOP, PPD, CAL) and microbiological (*P. g.*, *P. i.*, *F. n.*) at baseline, 15 days, 1 month.	Test sites showed significantly greater reduction in all clinical parameters and lower microbial counts compared to control (*p* < 0.01).	Propolis as an adjunct to SRP is effective in improving clinical and microbiological parameters in chronic periodontitis.
Chava & Vedula 2013 [152]	Split-mouth, single-blind RCT	30 patients with chronic periodontitis; India.	1. Test: Site + SRP + Thermo-reversible green tea gel 2. Control: Contralateral site + SRP + Placebo gel	Adjunctive subgingival delivery of a sustained-release green tea catechin gel after Phase I therapy.	Clinical parameters (GI, PPD, CAL) assessed at baseline and 4 weeks. In vitro drug release was also tested.	- Improvement of GI, PPD, and CAL within both groups from baseline to 4 weeks. - The test group showed significantly greater improvement than the control in the delta of all parameters.	Adjunctive local drug therapy with thermo-reversible green tea gel is effective in reducing pocket depth and inflammation in patients with chronic periodontitis over 4 weeks.
Gottumukkala et al. 2013 [153]	Single-blind, pilot RCT	23 patients with chronic periodontitis; India.	1. CHX: 0.2% CHX irrigation + SRP 2. CUR: 1% CUR irrigation + SRP 3. Control: Saline irrigation + SRP	Subgingival irrigation with assigned solution as an adjunct to SRP, performed at multiple intervals.	Clinical (BOP, redness, PI, PPD) and microbiological (BANA test) parameters evaluated at baseline, 1, 3, and 6 months.	- At 1 month, CUR group showed better results in BOP and PPD reduction. - By 6 months, CHX group showed the best results, with a slight recurrence in the CUR group.	1% CUR irrigation has a mild to moderate benefit as an adjunct to SRP, with efficacy comparable to CHX at first but less sustained.
Mahendra et al. 2013 [154]	Placebo-controlled, RCT	40 subjects with chronic periodontitis; India	Group A (Exp.): 33 sites (SRP + spirulina gel); Group B (Ctrl.): 31 sites (SRP alone)	Subgingival delivery of spirulina gel vs. placebo after SRP.	Clinical parameters (PPD, CAL) at baseline and 120 days.	Group A showed significantly greater reduction in PPD and gain in CAL compared to Group B (*p* < 0.05).	Spirulina gel as an adjunct to SRP is effective in improving periodontal parameters with anti-inflammatory and antioxidative benefits.
Matesanz et al. 2013 [155]	Placebo-controlled, parallel, RCT	22 patients with residual/relapsing periodontitis; Spain	Test: 10 patients (SRP + Xan–CHX gel); Control: 12 patients (SRP + placebo gel)	Subgingival application of xanthan-based 1.5% CHX gel vs. placebo.	Clinical (PPD, CAL, BOP) and microbiological evaluation at baseline, 1, 3, 6 months.	Limited clinical improvement in test group (BOP reduction, increased shallow pockets), but no significant intergroup differences. Microbiological impact minimal.	Xan–CHX gel may slightly improve clinical outcomes in residual pockets, but significant added benefit over placebo was not demonstrated.
Patel et al. 2012 [156]	Double-blind, split-mouth RCT	20 patients with chronic periodontitis; India.	1. Group A: SRP alone 2. Group B: SRP + Topical Ozonated Olive Oil (OZO) 3. Group C: Ozonated Olive Oil Monotherapy 4. Group D: Chlorhexidine Gel Monotherapy	Subgingival application of ozonated olive oil, either as an adjunct to SRP or as a monotherapy.	Clinical (PI, GI, SBI, PPD, CAL) and microbiological (Total bacterial counts & PCR for 8 pathogens) parameters assessed over 8 weeks. Patient-centered outcomes VAS recorded.	- Groups B and C showed significant improvement in all clinical and microbiological parameters over time. - Group B (adjunct) was most effective. Increased dentinal hypersensitivity was reported in Group B.	Ozonated olive oil is an effective adjunct to SRP and as a monotherapy for improving periodontal health. However, its use with SRP may cause increased dentinal hypersensitivity.
Coutinho A. 2012 [101]	RCT	20 patients with chronic periodontitis; India.	1. Group A: SRP + Propolis Extract Irrigation 2. Group B: SRP + Placebo Irrigation 3. Group C: SRP alone	Subgingival irrigation with a 20% hydroalcoholic propolis extract as an adjunct to SRP, twice a week for 2 weeks.	Clinical parameters (PI, GI, PPD, BOP, CAL) and microbiological samples (total anaerobic counts, *P. g.* levels) collected at multiple intervals over 8 weeks.	- Group A showed a significant decrease in total anaerobic counts and an increase in sites with low levels of *P. g*. - A significantly greater reduction in BOP and PPD was also seen in Group A.	Subgingival irrigation with propolis extract as an adjuvant to SRP is more effective than SRP alone or with a placebo, leading to significant clinical and microbiological improvement.
Rassameemasmaung et al., 2008 [157]	Single-blind RCT	- n = 31 adults with chronic periodontitis. - Systemically healthy, non-smokers. Thailand.	1. Test Group (n = 16): SRP + subgingival application of GM gel. 2. Control Group (n = 15): SRP alone.	Intervention: Subgingival application of *Garcinia mangostana* L. (mangosteen) pericarp gel (GM gel) into periodontal pockets immediately after SRP and again at 1 week. No placebo gel was used for the control group.	Clinical parameters (PPD, CAL, BOP, GI, PI) and microbiological analysis (phase-contrast microscopy) assessed at baseline, 1 month, and 3 months.	- Test Group had significantly greater PPD reduction than control at 3 months (shallow sites) and at 1 & 3 months (deep sites). - Test Group had significantly greater improvements in GI and BOP at the 3-month. - Microbiologically, the test group had a significantly higher percentage of cocci.	Topical application of GM gel as an adjunct to SRP enhances clinical outcomes, particularly in reducing probing depth, gingival inflammation, and bleeding on probing, likely due to its anti-inflammatory and antimicrobial properties.
**(B). Systemic supplementation summary of the included studies in this systematic review**							
**Author/Year**	**Study Design**	**Population/Study Location**	**Study Groups**	**Intervention/Exposure**	**Methods**	**Main Findings**	**Conclusion**
Lucchesi et al., 2025 [61]	Parallel-group RCT	38 smoking patients with periodontitis; Brazil	1. Test: Full-mouth ultrasonic debridement (FMUD) + Systemic RSV (500 mg/day) 2. Control: FMUD + Systemic Placebo	Systemic administration of RSV capsules for 180 days as an adjunct to non-surgical therapy.	Clinical parameters, microbiological analysis (PCR for *A. a.*, *T. f.*, *P. g*.), and immunoinflammatory markers (GCF cytokines via Luminex) assessed at baseline, 3, 6, and 12 months.	- RSV group had significantly lower PPD, CAL, and plaque scores over 12 months. - Lower counts of *A. a.* in deep sites at 6 months. - Lower levels of IL-1β (at 3 months) and IL-6 (at 3 & 12 months) in the RSV group.	Systemic resveratrol adjunctive to SRP improves clinical outcomes, reduces specific pathogen load, and modulates the immunoinflammatory response in periodontitis patients who smoke.
Farahmand et al., 2024 [158]	Double-blind, RCT	75 patients with chronic periodontitis; Iran	1. Group A: CoQ10 2. Group B: Omega-3 3. Group C: control	Daily CoQ10 (30 mg) or ω-3 (200 mg) for 2 months + SRP.	Clinical parameters (GI, PPD, CAL, PI, BOP) and salivary TAC measured at baseline and 2 months.	ω-3 group showed significantly greater reduction in GI and BOP vs. CoQ10 and control. All groups improved in PPD and CAL. TAC levels changed significantly in all groups.	ω-3 and CoQ10 supplements may improve periodontal health and antioxidant capacity, with ω-3 showing stronger anti-inflammatory effects.
Shirodkar et al., 2024 [159]	Triple-blind, placebo-controlled, RCT	80 chronic periodontitis patients; India	1. Test group: hesperidin; 2. Control group: placebo	Hesperidin (500 mg/day) or placebo for 3 weeks + SRP; follow-up at 3 and 6 weeks.	GI, SBI, PPD, CAL, and serum CRP measured.	Hesperidin significantly reduced serum CRP levels but did not significantly improve clinical parameters vs. placebo.	Hesperidin shows systemic anti-inflammatory effects but no significant short-term clinical benefit in periodontitis.
Ashrafzadeh et al., 2024 [89]	Parallel Pilot RCT	41 patients with T2DM and Periodontal Disease; Iran	1. C: Control (SRP only, n = 12) 2. I1: Omega-3 (n = 10) 3. I2: Cranberry Juice (n = 9) 4. I3: Cranberry Juice + Omega-3 (n = 10)	Oral Consumption (8 weeks): I1: 1 g ω-3 capsule, twice daily. I2: 200 mL Cranberry juice, twice daily. I3: 200 mL Cranberry juice enriched with 1 g ω-3, twice daily. All groups received SRP.	Clinical: PPD, CAL, BOP, and PI Biochemical (Serum/Saliva): TAC, MDA, Uric Acid, hs-CRP, IL-6, TNF-α. Timing: Baseline and after 8 weeks.	- I3 Group: Significant increase in serum and salivary TAC; significant decrease in salivary MDA, serum hs-CRP, and IL-6 vs. control. - I2 & I3 Groups: Significant decrease in serum MDA. - All Groups: Significant decrease in PPD and CAL post-intervention.	Consumption of cranberry juice enriched with ω-3 as an adjunct to non-surgical therapy can reduce systemic inflammation and oxidative stress, and improve periodontal status in diabetic patients with periodontal disease.
Laky et al., 2023 [109]	Double-Blind, Placebo-RCT	42 patients with Stage III/IV periodontitis; Austria	1. Test Group: SRP + Multinutrient Supplement 2. Control Group: SRP + Placebo	Oral multinutrient supplement (Vitamins C, E, Zinc, Selenium, Alpha-Lipoic-Acid, Cranberry, Grapeseed extract, CoQ10) taken twice daily for 2 months alongside nonsurgical therapy.	clinical parameters (PPD, BOP) assessed at baseline and 8-week reevaluation.	- The supplement group had a significantly greater reduction in PPD and BOP compared to placebo. - All parameters improved in both groups, with most showing greater improvement in the supplement group.	Multinutrient supplementation provided some additional benefit (in PPD and BOP reduction) as an adjunct to nonsurgical periodontal therapy, but the clinical relevance requires further exploration.
Nikniaz et al., 2023 [62]	Double-blind, placebo-RCT	40 patients with chronic periodontitis; Iran	1. Case Group (n = 20): SRP + RSV 2. Control Group (n = 20): SRP + Placebo (starch)	480 mg RSV daily (2 capsules) for 4 weeks, adjunct to SRP.	Clinical Parameters: PPD, CAL, PI, BOP. Inflammatory Markers: Salivary IL-8 and IL-1β (measured by ELISA). Timing: Measured at baseline and 4 weeks.	- PI showed a significant decrease in the RSV group vs. control (*p* = 0.0001). - PPD, CAL, BOP, IL-8, and IL-1β improved in both groups from baseline, but there were no significant differences between the groups.	RSV supplementation was helpful as an anti-inflammatory food supplement along with non-surgical periodontal treatment, with a significant effect on plaque reduction.
Stańdo-Retecka et al., 2023 [110]	Parallel-arm RCT	40 patients with untreated periodontitis stage III/IV; Poland	1. Test Group (n = 20): SRP + ω-3 PUFA (Fish Oil) 2. Control Group (n = 20): SRP alone	High-dose ω-3 PUFA (2.6 g EPA + 1.8 g DHA daily) as fish oil for 6 months, adjunct to SRP.	Clinical Parameters: PPD, CAL, REC, BOP, FMPS, rate of “closed pockets”. Microbiological: Counts of key periodontal pathogens (using PCR). Serum Analysis: Lipid profile via gas chromatography/mass spectrometry. Timing: Measured at baseline, 3 months, and 6 months.	- Short-term benefits (3 months): Test group had significantly lower BOP, higher CAL gain, and more “closed pockets,” especially in deep pockets (PPD ≥ 6 mm). - Long-term (6 months): Only BOP remained significantly lower in the test group. - Microbiological: Test group had significantly lower counts of all key periodontal pathogens at 6 months. - Serum: Confirmed increased n-3 PUFA and decreased n-6 PUFA levels in the test group.	Adjunctive high-dose ω-3 PUFA intake results in significant short-term clinical and microbiological benefits, and more effective pathogen clearance compared to SRP alone. It is safe and well-tolerated.
Nafade et al., 2022 [160]	Parallel-arm, RCT	60 subjects with mild to moderate chronic periodontitis; India	1. Test (n = 30): SRP + oolong tea; 2. Control (n = 30): SRP only	Test: SRP + daily intake of 4 g oolong tea for 30 days; Control: SRP only.	Clinical (GI, PI, PPD, CAL, BOP, LSI), biochemical (GPx, TAO, MDA in GCF, saliva, serum), microbiological (CFU of plaque bacteria) at baseline, 1, and 3 months.	Test group showed significant improvement in GI, antioxidant markers (GPx, TAO), reduction in MDA and CFU, maintained at 3 months. Control group improved at 1 month but deteriorated by 3 months.	Oolong tea as adjunct to SRP reduces oxidative stress and inflammation in chronic periodontitis, beneficial for patients with systemic conditions.
Maybodi et al., 2022 [112]	Double-blind, placebo RCT	30 patients with stage II–IV, grade B periodontitis; Iran	1. Intervention: ω-3 supplementation (1000 mg fish oil soft-gel daily) 2. Control: Placebo (soybean oil capsule)	Both groups received SRP and oral hygiene instructions; supplements taken for 3 months.	Clinical parameters (CAL, PPD, BOP) recorded at baseline and after 3 months.	- Significant reductions in CAL and PPD in ω-3 group vs. control (*p* = 0.001). - BOP decreased in both, but intergroup difference not significant.	ω-3 supplementation as an adjunct to SRP improved clinical periodontal outcomes, particularly CAL and PPD, supporting its anti-inflammatory potential.
Acharya et al., 2021 [113]	Double-blind, RCT	48 type 2 diabetic patients with chronic periodontitis; India	Test (SRP + GSE), Control (SRP + placebo)	200 mg GSE orally once daily for 8 weeks.	Clinical (PI, GI, PPD, CAL), metabolic (HbA1c, FBS), and biochemical (MPO, TAC) parameters at baseline, 3 and 6 months.	Significant improvement in TAC and MPO in test group at 3 and 6 months. Significant reduction in FBS at 3 months in test group. Clinical parameters improved in both groups, but only SBI and PI showed intergroup significance at 3 months.	GSE as adjunct to SRP reduces oxidative stress and inflammation, and improves glycemic control in diabetic patients with periodontitis.
Aslroosta et al., 2021 [161]	Preliminary RCT (Triple-blind)	44 patients; - Test: 15 completed - Control: 10 completed; Iran	1. SRP + Semelli (ANGIPARS) 2. SRP + Placebo	Semelli: 100 mg capsules (from Melilotus officinalis) taken twice daily for 3 months.	Clinical: PPD, CAL, MGI, mSBI, PI. Biochemical: GCF levels of IL-1β, 8-OHdG, LPO. Timing: Baseline and 3 months.	- Both groups improved significantly. Test group showed significantly greater improvement in MGI, mSBI, PPD, and CAL. - Biochemical parameters improved in both groups, but inter-group difference was not significant.	Systemic Semelli may be a beneficial adjunct for treating chronic periodontitis, improving clinical parameters. Larger, longer-term studies are needed.
Gholinezhad et al., 2020 [90]	Double-blind placebo-RCT	42 type 2 diabetic patients with chronic periodontitis; Iran	Intervention (ginger + SRP), Control (placebo + SRP)	2 g ginger supplement daily for 8 weeks alongside SRP.	Metabolic (HbA1c, FBG, lipid profile), oxidative (MDA, TAC), and periodontal (CAL, PPD, BOP, plaque) parameters at baseline and 8 weeks.	Significant reduction in HbA1c, FBG, MDA, CAL, and PPD in ginger group. HDL increased significantly. No significant changes in other lipids, TAC, plaque, or BOP.	Ginger supplementation with SRP improves glycemic control, antioxidant status, and periodontal parameters in diabetic patients with periodontitis.
Sravya et al., 2019 [94]	Single-blind, RCT	40 smokers with chronic periodontitis; India	1: SRP + Oxitard capsules; 2: SRP only	Oral Oxitard (herbal antioxidant, 2 capsules twice daily) for 3 months after SRP.	Serum procalcitonin levels (ELISA) and clinical parameters (GI, PPD, CAL) at baseline and 3 months.	Oxitard group showed greater reduction in procalcitonin, GI, PPD, and CAL vs. SRP alone.	Herbal antioxidant Oxitard as adjunct to SRP improves clinical and biochemical markers in smokers with periodontitis.
Zare Javid et al., 2019 [92]	Double-blind, placebo-RCT	42 T2DM patients with chronic periodontitis in Iran	1: Ginger (2 g/d) + SRP; 2: Placebo + SRP	Oral ginger supplementation (4 × 500 mg tablets daily) for 8 weeks alongside SRP.	Serum inflammatory (IL-6, TNF-α, hs-CRP), antioxidant (SOD, TAC, GPx), and periodontal (PPD, CAL, BOP, PI) measures baseline and 8 weeks.	Ginger significantly reduced IL-6, TNF-α, hs-CRP, PPD, CAL; increased SOD, GPx vs. placebo.	Ginger supplementation with SRP improves inflammatory, antioxidant, and periodontal status in diabetic periodontitis patients.
Javid et al., 2019 [91]	Double-blind, placebo-controlled RCT	43 type 2 diabetic patients with chronic periodontitis; Iran	1. Intervention: SRP + 480 mg/day RSV 2. Control: SRP + Placebo	Daily oral supplementation of 480 mg RSV for 4 weeks, adjunctive to SRP.	Serum levels of IL-6, TNF-α, TAC, and CAL measured at baseline and 4 weeks.	- Significant reduction in serum IL-6 within the RSV group. - No significant changes in TNF-α or TAC within or between groups. - CAL improved significantly in both groups post-SRP.	RSV supplementation may help reduce IL-6 but did not significantly affect TNF-α, TAC, or CAL beyond the improvements achieved with non-surgical periodontal therapy alone.
Taleghani et al., 2018 [162]	RCT	30 patients with chronic periodontitis; Iran	1. Control Group: 15 patients (SRP only) 2. Intervention Group: 15 patients (SRP + Green Tea)	Daily consumption of green tea herbal (2 times/day, after brushing) for 6 weeks.	Clinical parameters (PPD, BOP, and PI) measured at baseline and after 6 weeks. All patients received SRP at the start.	PPD and BOP: Significant reduction in both groups. The reduction was significantly greater in the green tea group. - PI: Significant reduction in both groups, but no significant difference between groups.	Daily consumption of green tea as a supplement to SRP can significantly improve probing depth and bleeding indices and can be used as an adjunct to enhance the effects of phase I therapy.
Babaei et al., 2018 [163]	Double-blind RCT	40 patients with chronic periodontitis; Iran	1. Control Group: 20 patients (Placebo capsule + SRP) 2. Intervention Group: 20 patients (Chicory capsule + SRP)	Oral supplementation with 1 g chicory leaf methanolic extract capsule (2 g daily) for 8 weeks.	Serum oxidative stress markers (TAC, MDA, uric acid), lipid profile (TC, TG, LDL, HDL), and PPD were assessed at baseline and after 8 weeks. All patients received non-surgical periodontal therapy.	- Oxidative Stress: TAC and uric acid increased, MDA decreased significantly in the intervention group. - Lipid Profile: TC, TG, and LDL decreased, while HDL increased in the intervention group. - PPD: Significant greater reduction in the chicory group.	Chicory leaf extract supplementation, as an adjunct to non-surgical periodontal therapy, improves periodontal status, likely through beneficial effects on systemic oxidative stress and lipid profiles.
Zare Javid et al., 2017 [164]	Double-blind, Placebo RCT	43 Type 2 Diabetic patients with chronic periodontitis; Iran	1. Intervention: SRP + 480 mg/day 2. RSV (21 pts) 3. Control: SRP + Placebo (22 pts)	Oral supplementation with 480 mg/day RSV capsules or placebo for 4 weeks, alongside SRP.	Fasting blood glucose, insulin, HOMA-IR, triglycerides, and PPD were measured at baseline and 4 weeks.	- Intervention group had significantly lower fasting insulin, HOMA-IR, and PPD than the control group post-intervention (*p* < 0.05). - No significant differences in FBG or triglycerides.	RSV supplementation as an adjunct to SRP may be beneficial for improving insulin resistance and periodontal status in diabetic patients with periodontitis.
Chopra et al., 2016 [165]	RCT	120 subjects with mild-moderate chronic periodontitis; India	1. Case Group (n = 60): SRP + Green Tea 2. Control Group (n = 60): SRP + Placebo	Oral intake of green tea sachets (2 cups/day for 12 weeks) vs. placebo after full-mouth SRP.	Clinical parameters (GI, PI, PPD, CAL, BOP) and total antioxidant capacity (FRAP assay) in GCF and plasma measured at baseline, 1, and 3 months.	- 8-fold greater increase in GCF antioxidant capacity in case group. - 6–7 fold greater increase in antioxidant capacity. - Significantly greater improvement in all clinical parameters in the case group.	Green tea intake is a promising adjunct to SRP, significantly increasing antioxidant levels and improving clinical outcomes in chronic periodontitis.
El-Sharkawy et al., 2016 [70]	Parallel masked and controlled randomized RCT	50 patients with T2DM and chronic periodontitis; Egypt	1. Placebo + SRP (n = 26) 2. Propolis + SRP (n = 24)	Oral supplementation of 400 mg propolis capsule once daily for 6 months vs. placebo, adjunctive to SRP.	HbA1c (primary outcome), FPG, serum CML, and periodontal parameters (PPD, CAL, GI, PI, EIBI) measured at baseline, 3, and 6 months.	- Propolis group had significant reductions in HbA1c (−0.96%), FPG, and CML. - Both groups showed improved periodontal health. - Propolis group had greater PPD reduction and CAL gain.	Propolis is a viable adjunct to SRP, improving glycemic control (HbA1c, FPG, CML) and periodontal outcomes in patients with T2DM and periodontitis.
Deore et al., 2014 [166]	Double-blind, Placebo RCT	60 systemically healthy patients with moderate/severe chronic periodontitis; India	1. Test: SRP + Septilin 2. Control: SRP + Placebo	Septilin: Oral systemic administration (2 capsules twice daily for 3 weeks). A polyherbal immunomodulator containing *Balsamodendron mukul*, *Tinospora cordifolia*, etc.	Clinical parameters (PI, GI, SBI, PPD, CAL) and CRP measured at baseline, 3 weeks, and 6 weeks.	- Test group showed significantly greater improvement in GI (at 3 weeks), SBI, and PPD (at 3 and 6 weeks). - No significant inter-group differences in CAL, PI, or CRP, though greater reduction was seen in the test group.	Dietary supplementation with the herbal immunomodulator Septilin may be a promising adjunct to SRP, aiding in improving clinical periodontal outcomes, particularly inflammation and probing depth.
Arora et al. 2013 [167]	Double-blind, placebo-RCT	42 patients with chronic periodontitis; India	Test: 21 (SRP + 8 mg lycopene/day); Control: 21 (SRP + placebo)	Systemic lycopene supplementation (8 mg/day) for 2 months.	Clinical (PI, MGI, BOP, PPD, CAL) and immunological markers (IL-1β, TNF-α, uric acid) at baseline and 2 months.	Test group showed significantly better improvement in PI, MGI, BOP, salivary IL-1β, and uric acid (*p* < 0.05). PPD, CAL, TNF-α showed non-significant improvement.	Lycopene supplementation adjunct to SRP improves gingival inflammation and antioxidant status, but longer studies are needed.
Chapple et al., 2012 [168]	Double-blind, placebo RCT	- n = 61 (60 completed) adults with chronic periodontitis. - Non-smokers, medically healthy, nutritionally replete. United Kingdom.	1. FV Group (n = 20): Fruit/vegetable juice powder concentrate. 2. FVB Group (n = 20): Fruit/vegetable/berry juice powder concentrate. 3. Placebo Group (n = 20): Microcrystalline cellulose.	Intervention: Daily oral capsules taken for 9 months, starting simultaneously with non-surgical periodontal therapy (scaling and root planing). Product: Juice Plus+^®^ capsules.	Clinical outcomes (PPD, CAL, BOP, and PI) assessed at baseline, 2-, 5-, and 8 months post-therapy. Adherence checked via capsule count, diary, and serum β-carotene levels. Intention-to-treat analysis.	- FV Group had significantly greater PPD reduction vs. placebo at 2 months. - FV Group had significantly lower BOP at 5 months and lower plaque scores at 8 months. - FVB Group showed greater reduction in GCF volume at deep sites at 2 months. - Benefits in the FV group were not sustained at 8 months.	Adjunctive supplementation with fruit/vegetable juice powder concentrate can improve initial clinical outcomes (pocket depth, bleeding, plaque) even in nutritionally replete patients, but effects may not be long-lasting.
El-Sharkawy et al., 2010 [169]	Double-masked, parallel-design, RCT	80 subjects with advanced chronic periodontitis; Egypt	Control: SRP + placebo; ω-3 + ASA: SRP + fish oil (900 mg EPA/DHA) + 81 mg aspirin daily	Daily dietary supplementation for 6 months following SRP.	Clinical parameters (PI, GI, BOP, PPD, CAL) and salivary biomarkers (RANKL, MMP-8) at baseline, 3, and 6 months.	ω-3 + ASA group had significantly greater PPD reduction & CAL gain vs. control. Salivary RANKL and MMP-8 levels reduced significantly more in the test group. Greater shift to healthy pockets (PPD < 4 mm).	Dietary supplementation with omega-3 PUFA and low-dose aspirin is a promising host modulatory therapy that augments clinical and biochemical outcomes of non-surgical periodontal treatment.
**(C). Rinse/topical adjuncts summary of the included studies in this systematic review**							
**Author/Year**	**Study Design**	**Population/Study Location**	**Study Groups**	**Intervention/Exposure**	**Methods**	**Main Findings**	**Conclusion**
Mohammed et al., 2025 [108]	Double-blind RCT	57 patients with periodontitis; Iraq	1. Test: RSV mouthwash 2. Positive Control: 0.12% Chlorhexidine (CHX) 3. Negative Control: Placebo	All groups received SRP. Adjunct mouthwash used twice daily for 60 sec for 4 weeks.	Clinical parameters (PI, BOP, PPD, CAL) assessed at baseline, 7, and 30 days. Salivary IL-6 and RANKL measured via ELISA. Patient feedback via VAS questionnaire.	- RSV & CHX were significantly better than placebo in reducing PI, BOP, PPD (but not CAL). - RSV & CHX significantly reduced IL-6 - All groups reduced RANKL, without differences.	RSV-containing mouthwash is an effective alternative to chlorhexidine as an adjunct to SRP for periodontitis.
Dzampaeva et al., 2021 [170]	Randomized controlled clinical trial RCT	60 patients - Group 1: 20 healthy controls - Group 2: 20 periodontitis patients - Group 3: 20 periodontitis patients; Russia	1. Control (Healthy) 2. SRP alone 3. SRP + Complex Phytoadaptogens (CFA)	CFA Cocktail: 70% alcoholic extract of Glycyrrhiza glabra and 40% extracts of Rhodiola rosea and Acanthopanax senticosus (2:1:1). Dosage was chronotype-based for 28 days.	Clinical: OHI-S, SBI, PI. Microcirculation: Doppler ultrasound (S, D, M velocities, PUI, RI). Timing: Baseline, post-treatment, 6 months.	- Group 3 showed significantly better improvement in OHI-S, SBI, and PI at 6 months vs. Group 2. - Microcirculation parameters (S, D, M, RI) were significantly closer to healthy controls in Group 3 at 6 months.	Chronotherapy with CFA as an adjunct to SRP is effective for long-term remission due to immunomodulatory, anti-inflammatory, antioxidant, and stress-limiting effects.
Anusha et al., 2019 [171]	Triple-blinded RCT	45 female RA patients with chronic periodontitis; India	1. Group A: SRP + 0.2% Chlorhexidine mouthwash 2. Group B: SRP + Mouthwash (Essential Oils + Curcumin) 3. Group C: SRP alone	Use of assigned mouthwash (10 mL, 1 min, twice daily) for 6 weeks as an adjunct to SRP.	Periodontal (PI, PPD, CAL) and RA activity (ESR, CRP, RF, ACPA) parameters measured at baseline and 6 weeks.	- All groups showed significant improvement in all parameters. - Plaque & RA markers: Highest % reduction in Group B. - PPD and CAL: Highest % reduction in Group A. - All inter-group differences in % reduction was significant.	Mouthwash containing essential oils and curcumin is effective as an adjunct to SRP, significantly reducing the disease activity of both rheumatoid arthritis and chronic periodontitis.
Azad et al. 2016 [172]	Double-blind, RCT	46 patients with moderate chronic periodontitis; Germany	Test: 23 (SRP + essential oil mouthwash); Control: 23 (SRP + placebo)	Essential oil mouthwash (Cymbopogon, Thymus, Rosmarinus) for 14 days post-SRP.	Clinical (PPD, CAL, BOP) and microbiological analysis at baseline, 3 and 6 months.	Test group showed significantly better CAL and BOP reduction, and greater reduction in *T. d.* and *F. n.*	Adjunctive essential oil mouthwash improves clinical and microbiological outcomes in moderate chronic periodontitis.

**Abbreviations for (A):** *A. a.*: *Aggregatibacter actinomycetemcomitans*; AOEOO: activated ozonated extra-virgin olive oil; BANA: N-benzoyl-DL-arginine-β-naphthylamide; BAPNA: Nα-benzoyl-DL-arginine-p-nitroanilide; BOP: bleeding on probing; BPB: black pigment producing microorganisms; CAL: clinical attachment level; CHX: chlorhexidine; CUR: curcumin; EGB: ginkgo biloba ex-tract; ELISA: enzyme-linked immunosorbent assay; FMPS: full-mouth plaque score; *F. n.*: *Fusobacterium nucleatum*; GBI: gingival index bleeding; GCF: gingival crevicular fluid; GI: gingival index; GM: *Garcinia mangostana*; GSE: grape seed extract; HPLC: high-performance liquid chromatography; IBD: intrabony defect; LDD: local drug delivery; MA: morus alba; MDA: malondialdehyde; MGI: modified gingival index; MMP-8: matrix metalloproteinase-8; mSBI: modified sulcus bleeding index; OHI: oral hygiene index; PBI: papilla bleeding index; PCR: Polymerase Chain Reaction; PCT: procalcitonin; *P. i.*: *Prevotella intermedia*; PI: plaque index; PDT: photodynamic therapy; *P. g.*: *Porphyromonas gingivalis*; PGA/MB/AV: poly-L-glycolic acid nanoparticles loaded methylene blue in *Aloe vera* gel; PPD: probing pocket depth; RAL: relative attachment level; RCT: randomized clinical trial; SBI: sulcus bleeding index; SLMs: solid lipid microparticles; SOD: superoxide dismutase; SRP: scaling and root planing; TAC: total antioxidant capacity; *T. d.*: *Treponema denticola*; *T. f.*: *Tannerella forsythia*; TGF-β1: transforming growth factor beta 1; TNF-α: tumor necrosis factor-alpha; T2DM: type 2 diabetes mellitus; VAS: visual analogue scale; *V. p.*: *Veillonella parvula*. **Abbreviations for (B):** *A. a.*: *Aggregatibacter actinomycetemcomitans*; ASA: aspirin; BOP: bleeding on probing; CAL: clinical attachment level; CFU: colony forming unit; CML: Nε-(carboxymethyl) lysine; CRP: C-Reactive Protein; DHA: docosahexaenoic acid; EIBI: Eastman interdental bleeding index; ELISA: enzyme-linked immunosorbent assay; EPA: eicosapentaenoic acid; FBG, fasting blood glucose; FBS: fasting blood sugar; FMPS: full-mouth plaque score; FMUD: full-mouth ultrasonic debridement; FPG: fasting plasma glucose; FRAP: ferric reducing antioxidant power; GCF: gingival crevicular fluid; GI: gingival index; GPx: glutathione peroxidase; GSE: grape seed extract; HbA1c: glycated hemoglobin; HDL: high-density lipoprotein cholesterol; HOMA-IR: homeostatic model assessment of insulin resistance; hs-CRP: high-sensitivity C-reactive protein; LDL: low-density lipoprotein cholesterol; LPO: lipid peroxidation; LSI: lobene stain index; MDA: malondialdehyde; MGI: modified gingival index; MMP-8: matrix metalloproteinase-8; MPO: myeloperoxidase; mSBI: modified sulcus bleeding index; PCR: Polymerase Chain Reaction; PI: plaque index; *P. g.*: *Porphyromonas gingivalis*; PPD: probing pocket depth; PUFA: polyunsaturated fatty acids; RCT: randomized clinical trial; REC: gingival recession; RSV: Resveratrol; SBI: sulcus bleeding index; SOD: superoxide dismutase; SRP: scaling and root planing; TAC: total antioxidant capacity; TAO: total antioxidants; TC: Total cholesterol; *T. f.*: *Tannerella forsythia*; TG: triglycerides; TNF-α: tumor necrosis factor-alpha; T2DM: type 2 diabetes mellitus; ω-3: omega-3; 8-OHdG: 8-hydroxy-2-deoxyguanosine. **Abbreviations for (C):** ACPA: Anti-Citrullinated Protein Antibody; BOP: bleeding on probing; CAL: Clinical attachment level; CFA: complex phytoadaptogens; CHX: chlorhexidine; CRP: C-Reactive Protein; D: diastolic; ELISA: enzyme-linked immunosorbent assay; ESR: Erythrocyte Sedimentation Rate; *F. n*: *Fusobacterium nucleatum*; M: mean; OHI-S: Simplified Oral Hygiene Index; PI: plaque index; PPD: probing pocket depth; PUI: pulsatility index (Gosling index); RA: Rheumatoid Arthritis; RCT: randomized clinical trial; RF: Rheumatoid Factor; RI: resistivity index (Pourcelot index); RSV: Resveratrol; S: systolic; SBI: sulcus bleeding index; SRP: Scaling and Root Planing; *T. d.*: *Treponema denticola*; VAS: visual analogue scale.

### 3.3. Synthesis of Results by Clinical Outcome

#### 3.3.1. Locally Delivery Therapies

Locally delivered adjunctive therapies constituted the largest and most intensively studied intervention category (Table 2A). These approaches included herbal gels, biodegradable chips, nanofiber scaffolds, and sustained-release carriers designed to deliver bioactive compounds directly into periodontal pockets. This strategy was widely used for curcumin, *Aloe vera*, propolis, green tea catechins, pomegranate extracts, tulsi, mangosteen, and a variety of botanical gels. This delivery strategy aims to achieve high local concentrations while minimizing systemic exposure, thereby targeting the subgingival microenvironment where inflammation and dysbiosis are concentrated.

Across trials, locally delivered natural adjuncts consistently enhanced short-term clinical outcomes when compared with SRP alone. Additional reductions in PPD were frequently reported, particularly in moderate-to-deep periodontal pockets, suggesting that local anti-inflammatory and antimicrobial actions may improve post-debridement healing. Several studies also demonstrated adjunctive CAL gains, although variability in measurement timing and baseline disease severity contributed to heterogeneous findings. Improvements in gingival indices and BOP were among the most reproducible outcomes, reflecting enhanced local inflammatory resolution (Table 2A).

A subset of investigations incorporated microbiological assessments, reporting reductions in pathogenic species such as *Porphyromonas gingivalis* and *Aggregatibacter actinomycetemcomitans*. While not all intergroup differences reached statistical significance, the general trend supported adjunctive suppression of microbial burden. Some trials also evaluated inflammatory or oxidative biomarkers, observing patterns consistent with reduced tissue inflammation. Despite variability in formulation, carrier materials, dosing protocols, and frequency of application, the overall direction of effect favored adjunctive benefit (Table 2A).

Clinical improvements associated with local delivery were most prominent during early follow-up intervals (1–3 months), with fewer studies extending observation beyond six months. The predominance of short-term evaluation suggests that locally delivered natural therapies primarily influence early healing dynamics and inflammatory modulation. Although encouraging, heterogeneity in delivery systems and trial design limits definitive conclusions regarding optimal formulations or sustained long-term efficacy (Table 2A).

#### 3.3.2. Systemic Delivery Therapies

Systemically administered natural adjuncts represented a distinct therapeutic strategy aimed at modulating host inflammatory and oxidative pathways rather than exerting direct localized antimicrobial effects. Interventions in this category included omega-3 polyunsaturated fatty acids, polyphenolic compounds, antioxidant-rich nutraceutical formulations, and related supplements intended to influence systemic inflammatory tone [59,61,62,70,72,89,91,92,98,108,109,110,111,112,113,114,115,116,146,154,157,165,169] (Table 2B). These systemic approaches were generally administered daily for 4 weeks to 12 months.

Across trials, systemic supplementation was associated with additional improvements in periodontal clinical parameters when combined with SRP. Adjunctive reductions in PPD and modest CAL gains were reported in several studies, alongside improvements in gingival inflammation indices. These effects were particularly evident in patient populations characterized by elevated systemic inflammatory burden, including individuals with metabolic dysregulation or smoking history, suggesting that systemic host modulation may contribute to enhanced periodontal healing (Table 2B).

Many studies in this category extended evaluation beyond purely clinical endpoints by assessing biochemical markers of inflammation and oxidative stress. Reductions in circulating or local inflammatory mediators, including IL-1β, TNF-α, and IL-6, as well as improvements in antioxidant profiles were reported in multiple trials. These findings provide biological context for the observed clinical benefits and support a plausible mechanism linking systemic modulation to periodontal tissue response (Table 2B). Studies involving diabetic patients also reported favorable metabolic effects, including reductions in HbA1c, fasting glucose, and HOMA-IR [61,93,94]. Nevertheless, considerable heterogeneity existed in supplement composition, dosing regimens, duration of administration, and baseline patient characteristics. Effect sizes were generally modest, and inter-study variability precluded firm conclusions regarding superiority of specific agents. As with local therapies, benefits were most consistently observed during short- to medium-term follow-up, with limited evidence regarding sustained long-term periodontal stabilization (Table 2B).

#### 3.3.3. Rinse-Based Interventions

Rinse-based and topical home-use formulations comprised a smaller but clinically relevant subset of adjunctive therapies, used particularly for herbal solutions, matcha preparations, goji berry extract, and propolis mouthwashes (Table 2C). These interventions included herbal mouthrinses, antioxidant solutions, and plant-derived extracts intended to complement mechanical plaque control and enhance gingival health through repeated topical exposure. Trials evaluating rinse-based adjuncts commonly reported improvements in superficial clinical parameters, particularly gingival inflammation indices, BOP, and plaque accumulation. These outcomes suggest that rinse formulations primarily influence supragingival biofilm dynamics and soft tissue inflammatory responses. Effects on deeper periodontal parameters, such as PPD and CAL, were more variable and generally less pronounced, reflecting the limited ability of rinse-based delivery to penetrate deep periodontal pockets (Table 2C).

Microbiological analyses, when performed, indicated reductions in bacterial load consistent with improved plaque control, although methodological diversity limited cross-study comparison. Variability in rinse composition, frequency of use, patient adherence, and duration of therapy likely contributed to heterogeneity in reported outcomes. Overall, rinse-based adjuncts appear to support gingival inflammation control and plaque management rather than serve as primary drivers of deep periodontal regeneration. Observed benefits were predominantly short-term, aligning with the transient nature of topical antimicrobial and anti-inflammatory effects. These findings underscore the role of rinse-based therapies as supportive adjuncts within comprehensive periodontal maintenance protocols (Table 2C).

### 3.4. Clinical Outcomes

When results were examined by clinical outcome rather than by intervention type, several consistent patterns emerged. PPD reduction was the most frequently reported and most consistently improved parameter across studies, particularly at short-term follow-up. CAL gains were also observed, although with greater variability and generally smaller effect sizes. Measures of gingival inflammation, including BOP and gingival indices, showed favorable responses in many trials, especially those employing locally delivered formulations. In contrast, microbiological and biochemical outcomes were reported less uniformly, and their results were more heterogeneous, limiting quantitative synthesis. This outcome-oriented perspective highlights that the primary adjunctive benefits of natural products relate to modulation of inflammation and pocket depth rather than to uniform effects across all periodontal endpoints.

Taken together, the stratified synthesis highlights consistent short-term adjunctive benefits across multiple delivery modalities, yet these findings must be interpreted within the context of substantial clinical and methodological variability. Differences in formulation, dosage, delivery systems, patient characteristics, and follow-up intervals limit direct cross-study comparability and contribute to observed heterogeneity in effect estimates. Accordingly, the pooled results should be understood as reflecting overall trends in adjunctive benefit rather than definitive evidence of superiority for any specific compound or delivery strategy. While the direction of effect generally favors adjunctive natural therapies in improving inflammatory parameters and PPD reduction, variability in study design and outcome reporting underscores that these results are best viewed as indicative rather than conclusive. This interpretive framework supports cautious translation of findings into clinical decision-making and reinforces the need for standardized, adequately powered trials to clarify the magnitude, durability, and mechanistic basis of adjunctive effects. Additionally, across interventions, treatment effects were more consistently detected at short- to medium-term follow-up intervals, typically within 1 to 6 months after therapy. Fewer studies extended follow-up beyond this period, and long-term effects were therefore less certain. This temporal pattern underscores that current evidence primarily supports short-term adjunctive benefits, with limited data available on long-term periodontal stability. The strength of these findings should be interpreted in the context of the methodological quality and certainty of evidence assessments. (Q7).

### 3.5. Risk of Bias Analysis

**Figure 2:**
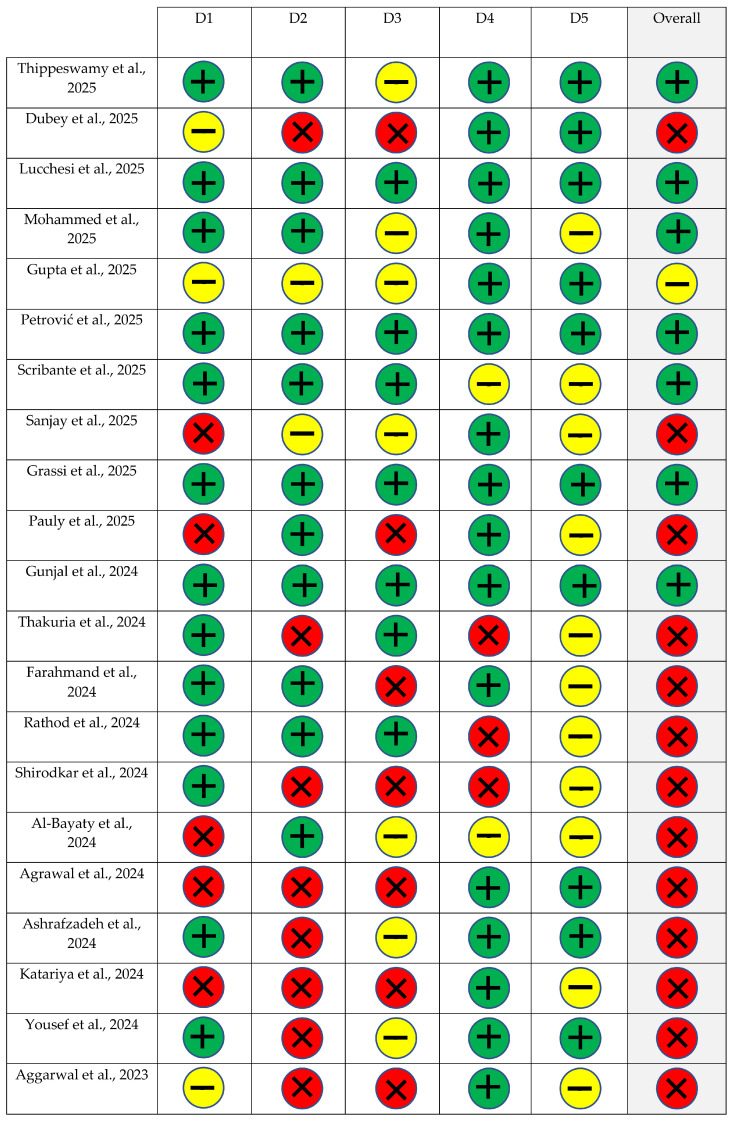

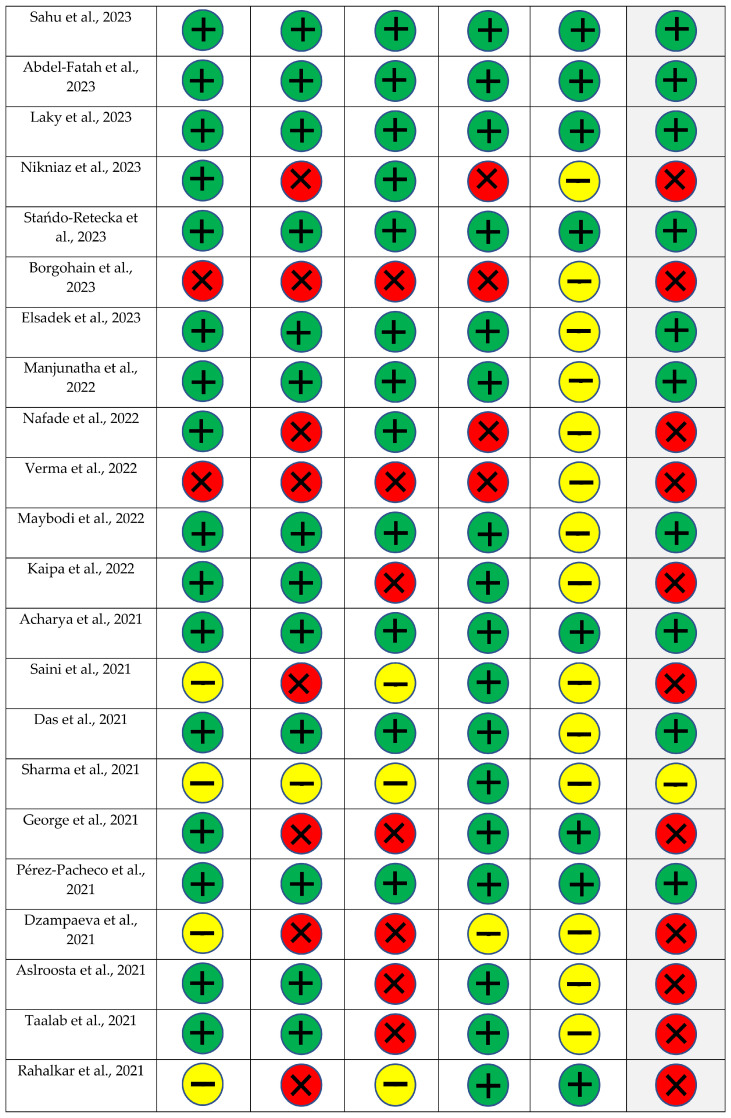

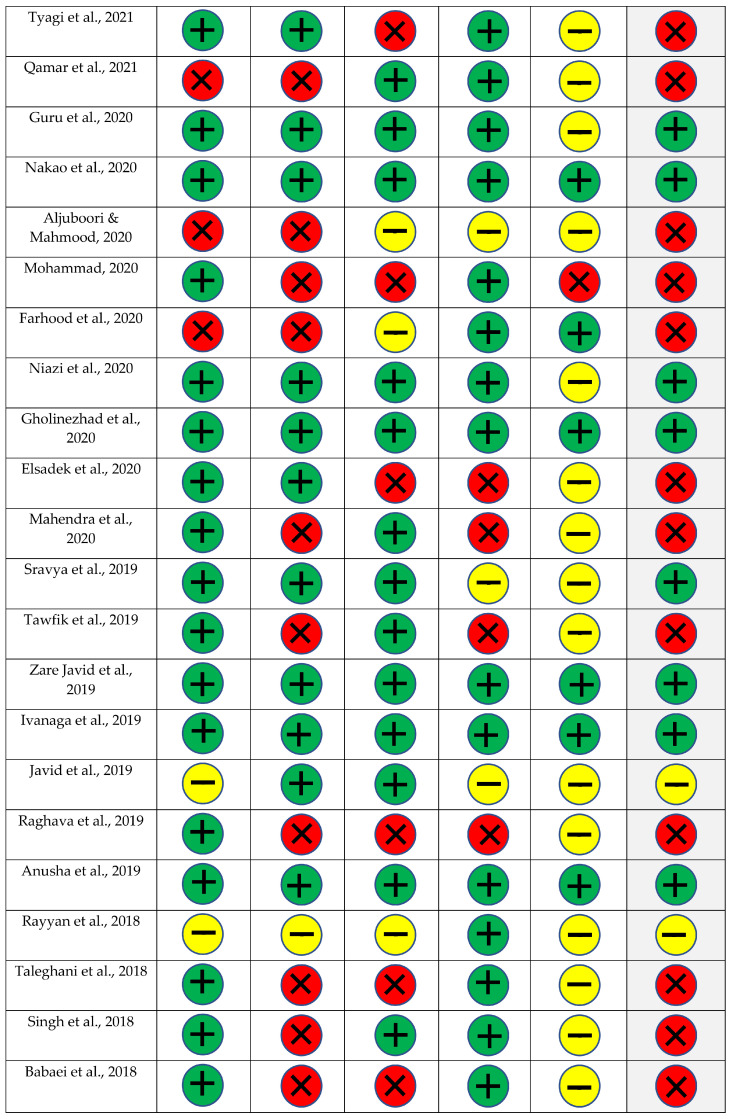

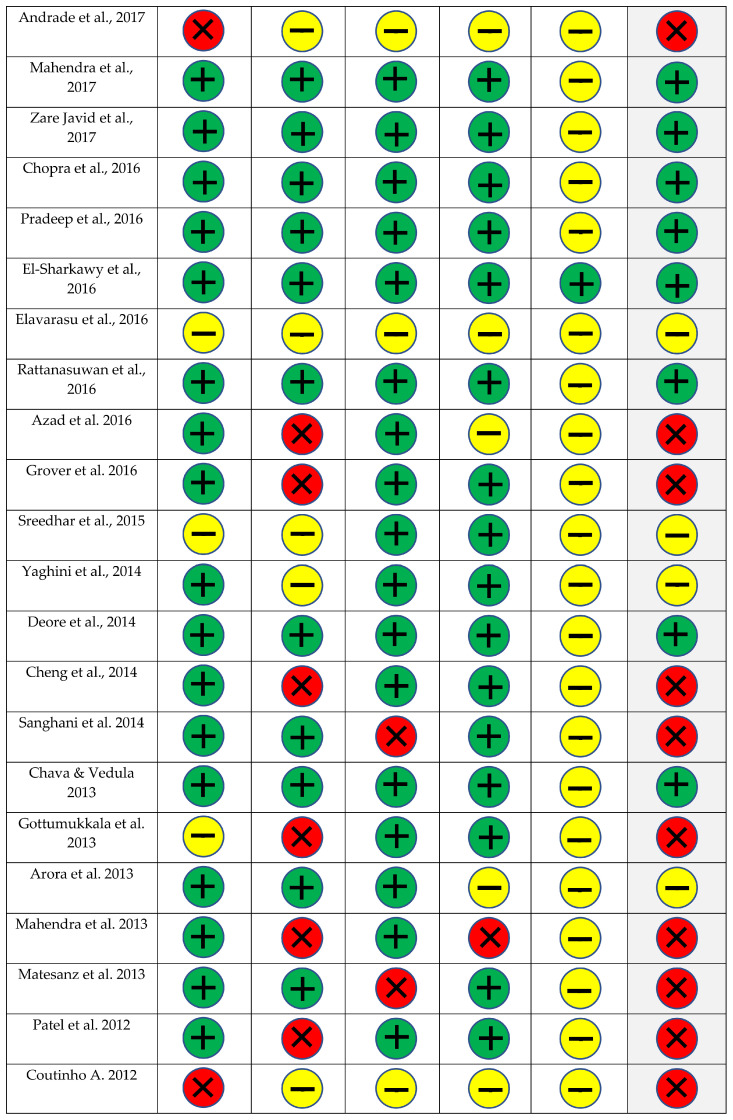

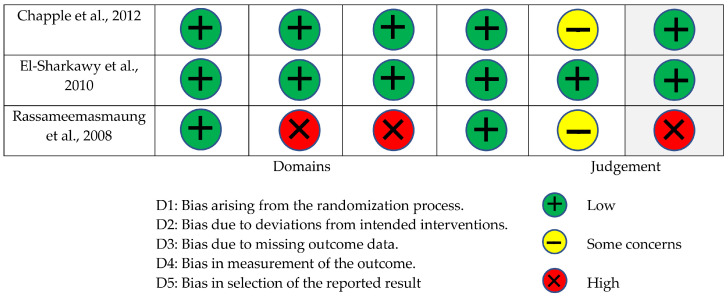
Risk of bias of the included studies [57,58,59,61,62,70,72,89,90,91,92,93,94,95,96,97,98,99,100,101,102,103,104,105,106,107,108,109,110,112,113,114,115,116,117,118,119,120,121,122,123,124,125,126,127,128,129,130,131,132,133,134,135,136,137,138,139,140,141,142,143,144,145,146,147,148,149,150,151,152,153,154,155,156,157,158,159,160,161,162,163,164,165,166,167,168,169,170,171,172].

The overall methodological quality of the included studies showed considerable variability, with most trials presenting either low risk of bias or some concerns, while a smaller proportion exhibited high risk in one or more domains (Figure 2). Assessment of the randomization process revealed that many studies reported adequate random sequence generation and allocation concealment; however, several trials provided insufficient information, leading to classification as having some concerns. A few studies demonstrated clear selection issues, resulting in a high-risk judgment in this domain.

With respect to deviations from the intended interventions, most studies were judged to be at low risk, particularly those employing blinded or double-blinded designs. Nevertheless, several studies lacked blinding of participants, investigators, or outcome assessors, creating uncertainty regarding performance or detection bias. A limited number of cases clearly showed deviations likely to have influenced clinical outcomes, resulting in a high-risk classification.

Missing outcome data were generally well handled, with most studies presenting complete follow-up or transparently addressing attrition. Only a few trials showed a high risk due to substantial losses to follow-up or inadequate explanation for missing data. Measurement of outcomes was frequently rated as low risk, since clinical periodontal parameters such as PPD, CAL, PI, and GBI are objectively recorded. However, studies that did not blind examiners or did not specify whether examiners were calibrated were considered to have some concerns. High-risk assessments in this domain mainly occurred in studies where outcome measurement procedures were unclear or inconsistent across groups.

Regarding selective reporting, the majority of trials adhered to their stated objectives and reported all prespecified outcomes. Some concerns were raised in studies with incomplete reporting of biochemical or microbiological data, or in studies in which the methods sections suggested additional outcomes that were not fully presented. Only a few studies showed clear evidence of selective outcome reporting and were therefore rated as high risk in this domain.

Considering all domains, the overall risk of bias across studies ranged from low to some concerns, with some categorized as high risk. Trials published more recently tended to exhibit better methodological rigor, particularly in randomization, blinding, and reporting transparency. Despite heterogeneity in study design and quality, the majority of included trials demonstrated acceptable internal validity, allowing meaningful interpretation of their findings in this systematic review.

### 3.6. Main Results and Meta-Analysis

The quantitative synthesis evaluated the adjunctive effects of several natural compounds when combined with SRP compared with SRP alone. The outcomes most consistently reported across studies were PPD, CAL, BOP, and PI, assessed at different follow-up intervals. Random- or fixed-effects models were applied according to the degree of statistical heterogeneity observed in each comparison.

**Figure 3:**
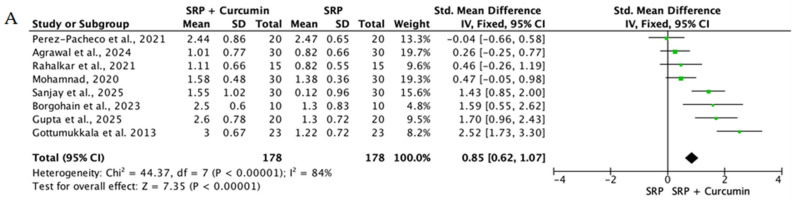

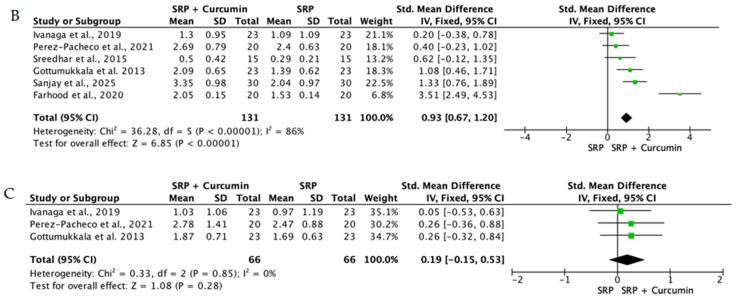
(**A**–**C**). Forest plot of the standardized mean difference (SMD) in PPD levels at 1 (**A**) [58,93,118,125,127,135,140,153], 3 (**B**) [58,116,118,141,149,153] and 6 (**C**) [58,116,153] months, respectively comparing the SRP + curcumin group with the SRP-only control group. Individual study effect sizes with 95% confidence intervals (CIs) are shown, with the size of the squares proportional to study weight, and the diamond represents the pooled effect estimate from a random-effects model. Positive SMD values favor SRP + curcumin, indicating a greater reduction in PPD levels compared with SRP alone. Heterogeneity among studies is reported using the χ^2^ test and I^2^ statistic.

**Figure 4:**
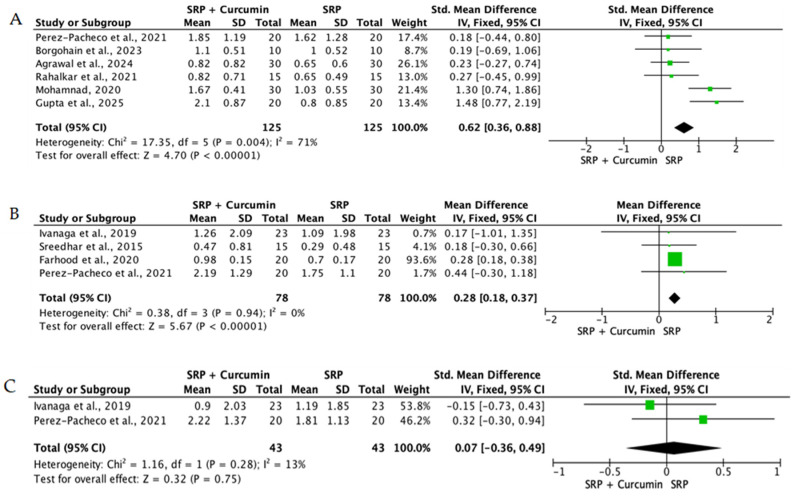
(**A**–**C**) Forest plot of the SMD in CALs at 1 (**A**) [58,93,105,127,135,140], 3 (**B**) [58,116,141,149], and 6 (**C**) [58,116] months, respectively, comparing the SRP + curcumin group with the SRP-only control group. Individual study effect sizes with 95% CIs are shown, with the size of the squares proportional to study weight, and the diamond represents the pooled effect estimate from a random-effects model. Positive SMD values favor SRP + curcumin, indicating a greater reduction in CAL levels compared with SRP alone. Heterogeneity among studies is reported using the χ^2^ test and I^2^ statistic.

**Figure 5:**
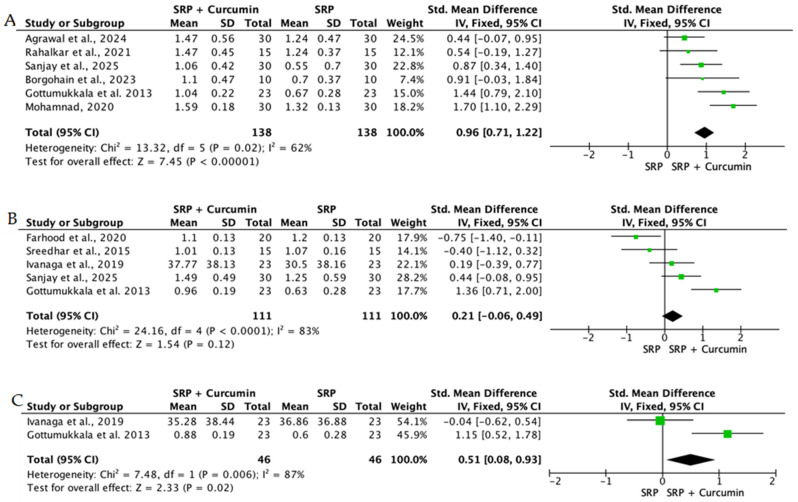
(**A**–**C**). Forest plot of the SMD in PI levels at 1 (**A**) [105,118,127,135,140,153], 3 (**B**) [116,118,141,149,153], and 6 (**C**) [116,153] months, respectively, comparing the SRP + curcumin group with the SRP-only control group. Individual study effect sizes with 95% CIs are shown, with the size of the squares proportional to study weight, and the diamond represents the pooled effect estimate from a random-effects model. Positive SMD values favor SRP + curcumin, indicating a greater reduction in PI levels compared with SRP alone. Heterogeneity among studies is reported using the χ^2^ test and I^2^ statistic.

For curcumin, adjunctive SRP use resulted in a statistically significant (*p* < 0.05) additional reduction in PPD at one (Figure 3A) and three months (Figure 3B). At one month, the pooled analysis demonstrated a greater PPD reduction of 0.85 mm in the test group, with the confidence interval indicating a robust effect despite significant heterogeneity (I^2^ = 84%). This effect was maintained at three months, with a pooled additional reduction of 0.93 mm in favor of the adjunctive curcumin group, again accompanied by heterogeneity among studies (I^2^ = 86%). By six months (Figure 3C), however, the pooled effect size was markedly attenuated, and no statistically significant difference (95% CI crossed zero) was noted, suggesting a waning clinical effect over time. In terms of CAL (Figure 4A–C), the pooled estimates at one and three months indicated greater attachment loss in the control group, reflecting a modest but statistically significant (*p* < 0.05) benefit for curcumin; heterogeneity was evident at one month but not at later time points. At six months, no statistically significant differences between groups were detected. PI analyses (Figure 5A–C) showed a statistically significant (*p* < 0.05) additional reduction in plaque accumulation in the curcumin group at one, three, and six months, while results at three months were inconclusive and heterogeneous.

**Figure 6:**
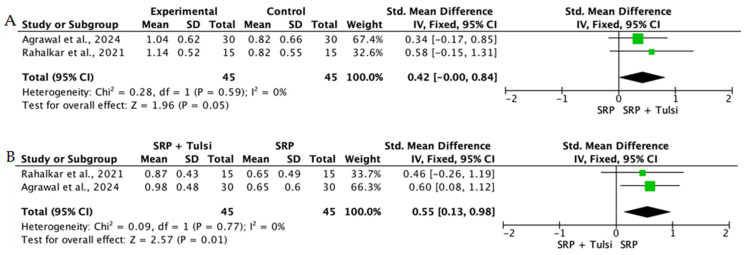

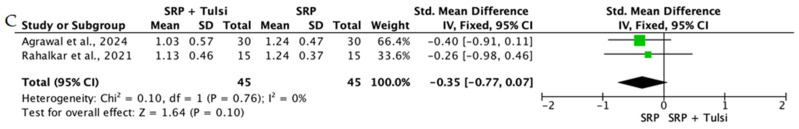
(**A**–**C**). Forest plot of the SMD in PPD (**A**), CAL (**B**), and PI (**C**) levels at 1 month, respectively, comparing the SRP + tulsi group with the SRP-only control group [105,135]. Individual study effect sizes with 95% CIs are shown, with the size of the squares proportional to study weight, and the diamond represents the pooled effect estimate from a random-effects model.

For tulsi, the available evidence was limited to short-term outcomes. The pooled analysis at one month showed a tendency toward greater PPD reduction in the adjunctive group (Figure 6A); however, the confidence interval included the null value, indicating no statistically significant difference (*p* < 0.05). CAL outcomes at one month (Figure 6B) favored the control group, with a statistically significant (*p* < 0.05) greater attachment loss observed in patients receiving SRP alone, suggesting a potential protective effect of tulsi. PI data (Figure 6C) at one month showed no statistically significant difference (95% CI crossed zero).

**Figure 7:**
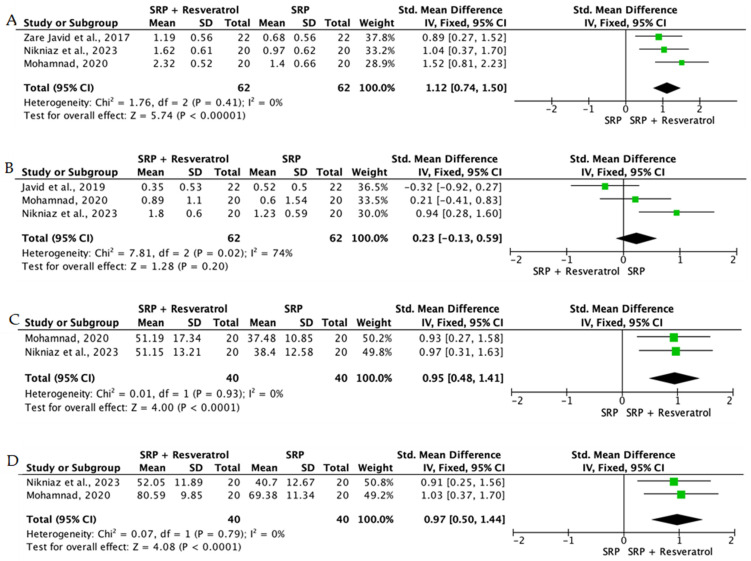
(**A**–**D**). Forest plot of the SMD in PPD (**A**) [62,140,164], CAL (**B**) [62,91,140], BOP (**C**) [62,140], and PI (**D**) [62,140] levels at 1 month, respectively, comparing the SRP + resveratrol group with the SRP-only control group. Individual study effect sizes with 95% CIs are shown, with the size of the squares proportional to study weight, and the diamond represents the pooled effect estimate from a random-effects model.

Adjunctive resveratrol demonstrated a consistent short-term benefit. At one month (Figure 7A), the pooled PPD analysis showed a pronounced additional reduction of more than 1 mm, favoring the test group, with a narrow confidence interval and no heterogeneity (95% CI crossed zero). CAL changes (Figure 7B) did not differ significantly between groups, and the corresponding analysis showed heterogeneity, suggesting variability in attachment-level responses across studies. BOP and PI (Figure 7C,D) were also significantly reduced in the resveratrol group, indicating improved control of gingival inflammation (95% CI crossed zero). PI outcomes similarly favored adjunctive resveratrol, with a significant reduction and homogeneous results.

**Figure 8:**
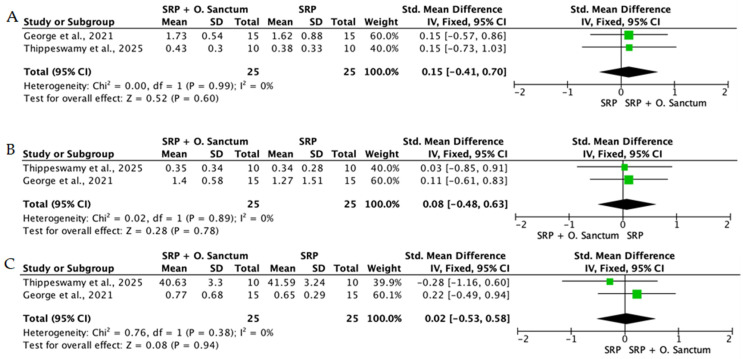
(**A**–**C**). Forest plot of the SMD in PPD (**A**), CAL (**B**), and BOP (**C**) levels at 1 month, respectively, comparing the SRP + O sanctum group with the SRP-only control group [102,133]. Individual study effect sizes with 95% CIs are shown, with the size of the squares proportional to study weight, and the diamond represents the pooled effect estimate from a random-effects model.

When *O. sanctum* was evaluated as an adjunct, pooled analyses at one month did not demonstrate a statistically significant difference (95% CI crossed zero) between groups for PPD, CAL, or BOP indices (Figure 8A–C). Statistical homogeneity (I^2^ = 0%) was detected, indicating a lack of measurable clinical benefit within the short follow-up period assessed.

**Figure 9:**
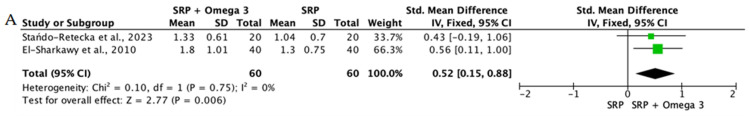

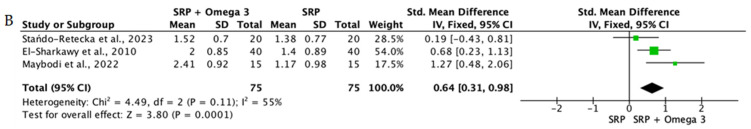
(**A**,**B**). Forest plot of the SMD in PPD levels at 1 (**A**) [110,169] and 3 (**B**) [110,112,169] months, respectively, comparing the SRP + Omega 3 group with the SRP-only control group. Individual study effect sizes with 95% CIs are shown, with the size of the squares proportional to study weight, and the diamond represents the pooled effect estimate from a random-effects model.

**Figure 10:**
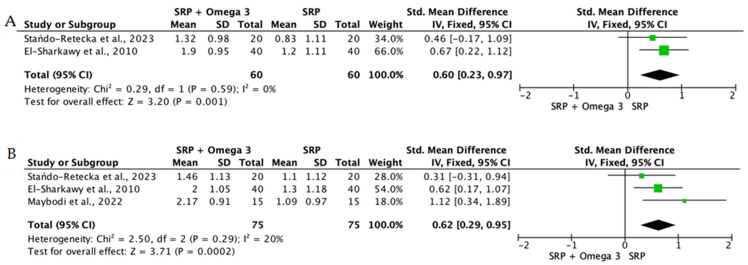
(**A**,**B**). Forest plot of the SMD in CALs at 1 (**A**) [110,169] and 3 (**B**) [110,112,169] months, respectively, comparing the SRP + Omega 3 group with the SRP-only control group. Individual study effect sizes with 95% CIs are shown, with the size of the squares proportional to study weight, and the diamond represents the pooled effect estimate from a random-effects model.

**Figure 11:**
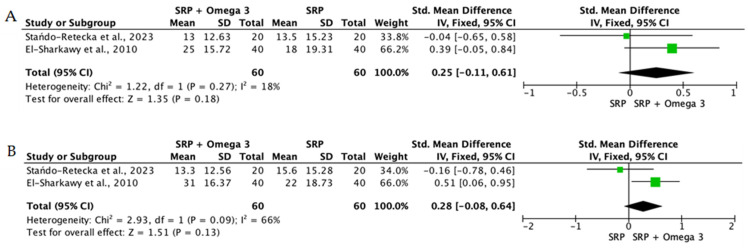
(**A**,**B**). Forest plot of the SMD in BOP levels at 1 (**A**) and 3 (**B**) months, respectively, comparing the SRP + Omega 3 group with the SRP-only control group [110,169]. Individual study effect sizes with 95% CIs are shown, with the size of the squares proportional to study weight, and the diamond represents the pooled effect estimate from a random-effects model.

**Figure 12:**
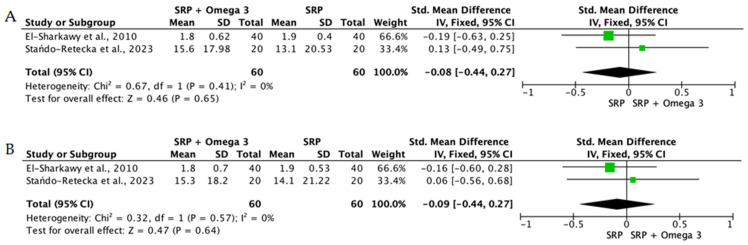
(**A**,**B**). Forest plot of the SMD in PI levels at 1 (**A**) and 3 (**B**) months, respectively, comparing the SRP + Omega 3 group with the SRP-only control group [110,169]. Individual study effect sizes with 95% CIs are shown, with the size of the squares proportional to study weight, and the diamond represents the pooled effect estimate from a random-effects model.

Omega-3 supplementation as an adjunctive therapy yielded significant improvements in PPD at both one (Figure 9A) and three months (Figure 9B) with pooled mean differences in moderate magnitude favoring the test group (*p* < 0.05). CAL outcomes consistently favored the test group at both time points, with greater attachment loss observed in patients receiving SRP alone, suggesting a beneficial effect of omega-3 supplementation on greater CAL gain in the adjunct group (*p* < 0.05) (Figure 10A,B). BOP showed a trend toward reduction in the omega-3 group, although the confidence intervals crossed the null value, and heterogeneity emerged at three months (*p* < 0.05) (Figure 11A,B). PI outcomes did not demonstrate significant benefits with adjunctive omega-3, with no significant heterogeneity detected (I^2^ = 0%) (Figure 12A,B).

**Figure 13:**
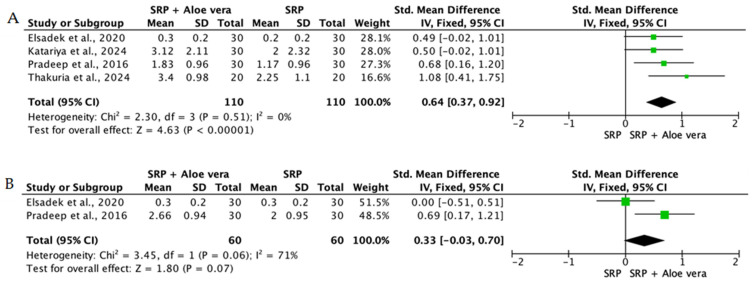
(**A**,**B**). Forest plot of the SMD in PPD levels at 3 (**A**) [72,98,122,126] and 6 (**B**) [72,98] months, respectively, comparing the SRP + *Aloe vera* group with the SRP-only control group. Individual study effect sizes with 95% CIs are shown, with the size of the squares proportional to study weight, and the diamond represents the pooled effect estimate from a random-effects model.

**Figure 14:**
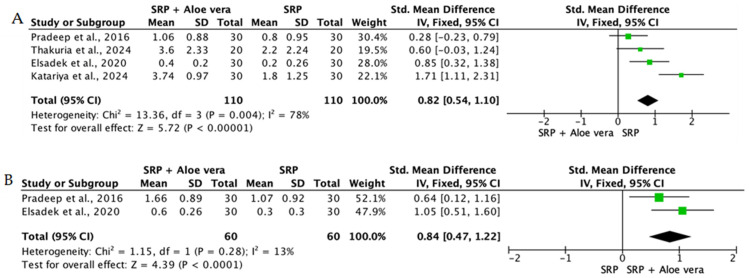
(**A**,**B**). Forest plot of the SMD in CALs at 3 (**A**) [72,98,122,126] and 6 (**B**) [72,98] months, respectively, comparing the SRP + *Aloe vera* group with the SRP-only control group. Individual study effect sizes with 95% CIs are shown, with the size of the squares proportional to study weight, and the diamond represents the pooled effect estimate from a random-effects model.

**Figure 15:**
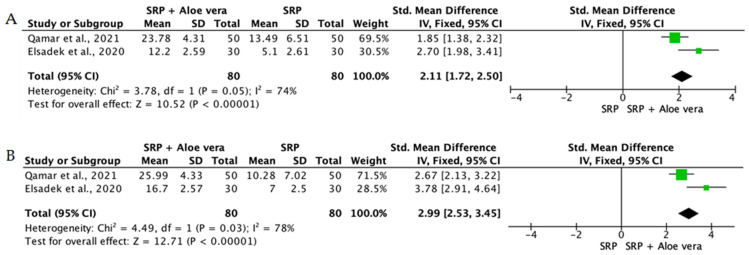
(**A**,**B**). Forest plot of the SMD in BOP levels at 3 (**A**) and 6 (**B**) months, respectively, comparing the SRP + *Aloe vera* group with the SRP-only control group [98,137]. Individual study effect sizes with 95% CIs are shown, with the size of the squares proportional to study weight, and the diamond represents the pooled effect estimate from a random-effects model.

**Figure 16:**
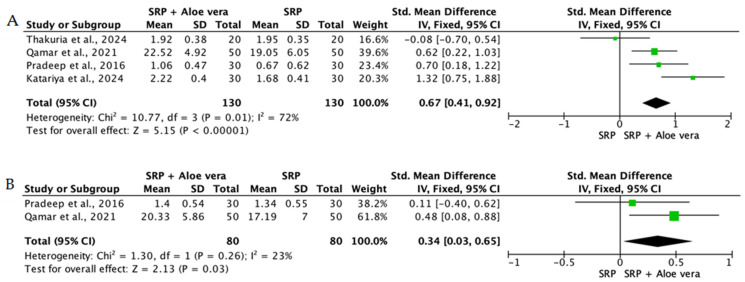
(**A**,**B**). Forest plot of the SMD in PI levels at 3 (**A**) [72,122,126,137] and 6 (**B**) [72,137] months, respectively, comparing the SRP + *Aloe vera* group with the SRP-only control group. Individual study effect sizes with 95% CIs are shown, with the size of the squares proportional to study weight, and the diamond represents the pooled effect estimate from a random-effects model.

For *Aloe vera*, significant adjunctive effects were observed primarily at intermediate time points. At three months, the pooled PPD analysis showed a significant additional reduction in favor of the *Aloe vera* group, with homogeneous findings (*p* < 0.05) (Figure 13A,B). At six months, the effect size decreased and lost statistical significance (*p* < 0.05), with heterogeneity detected. CAL analyses at both three and six months favored the control group, indicating greater CAL gain in the adjunctive group; heterogeneity was present at three months but not at six months (Figure 14A,B). BOP exhibited a marked, statistically significant (*p* < 0.05) reduction in the *Aloe vera* group at both three and six months, although heterogeneity was substantial, reflecting variability in effect magnitude (Figure 15A,B). PI analyses showed statistically significant (*p* < 0.05) reductions favoring adjunctive *Aloe vera* at both follow-up points, with heterogeneity at three months and homogeneity at six months (Figure 16A,B).

**Figure 17:**
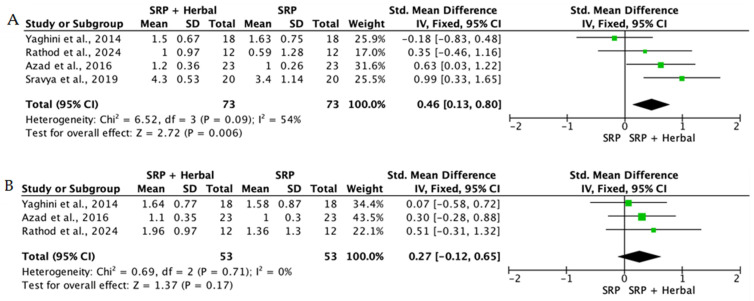
(**A**,**B**). Forest plot of the SMD in PPD levels at 3 (**A**) [94,123,150,172] and 6 (**B**) [123,150,172] months, respectively, comparing the SRP + Herbal group with the SRP-only control group. Individual study effect sizes with 95% CIs are shown, with the size of the squares proportional to study weight, and the diamond represents the pooled effect estimate from a random-effects model.

**Figure 18:**
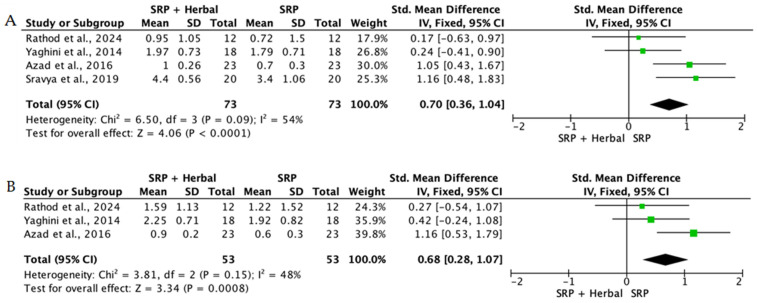
(**A**,**B**). Forest plot of the SMD in CALs at 3 (**A**) [94,123,150,172] and 6 (**B**) [123,150,172] months, respectively, comparing the SRP + Herbal group with the SRP-only control group. Individual study effect sizes with 95% CIs are shown, with the size of the squares proportional to study weight, and the diamond represents the pooled effect estimate from a random-effects model.

The pooled analysis of herbal formulations demonstrated modest but statistically significant (*p* < 0.05) additional PPD reduction at three months, with homogeneity (Figure 17A,B). By six months, the difference between groups demonstrated no significant heterogeneity (I^2^ = 0%). CAL outcomes at both three and six months favored the adjunctive group indirectly, as greater attachment loss was observed in the control group, and all analyses demonstrated significant homogeneity (*p* < 0.05) (Figure 18A,B).

**Figure 19:**
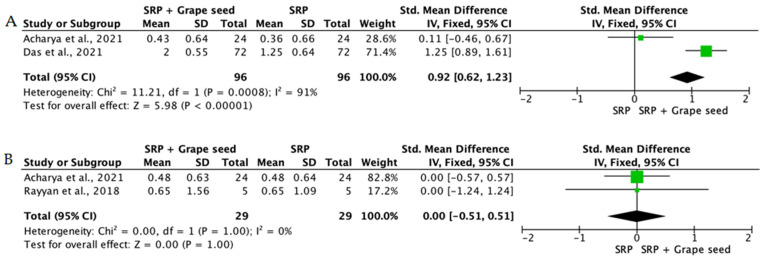
(**A**,**B**). Forest plot of the SMD in PPD levels at 3 (**A**) and 6 (**B**) months, respectively, comparing the SRP + Grape seed with the SRP-only control group [113,132]. Individual study effect sizes with 95% CIs are shown, with the size of the squares proportional to study weight, and the diamond represents the pooled effect estimate from a random-effects model.

**Figure 20:**
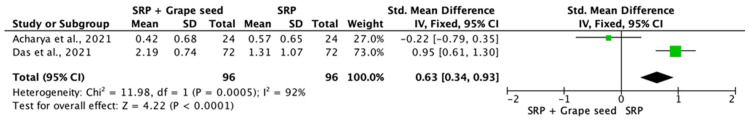
Forest plot of the SMD in CALs at 3 months comparing the SRP + Grape seed with the SRP-only control group [113,132]. Individual study effect sizes with 95% CIs are shown, with the size of the squares proportional to study weight, and the diamond represents the pooled effect estimate from a random-effects model.

**Figure 21:**
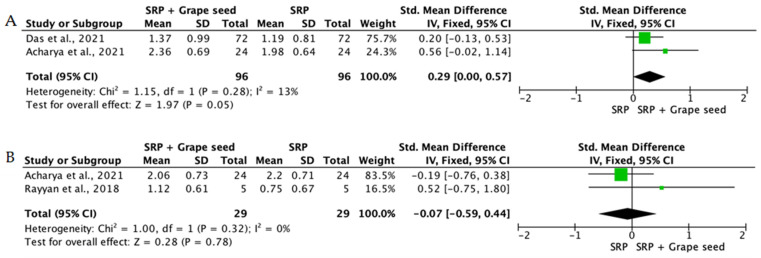
(**A**,**B**). Forest plot of the SMD in PI levels at 3 (**A**) [113,132] and 6 (**B**) [113,144] months, respectively, comparing the SRP + Grape seed with the SRP-only control group. Individual study effect sizes with 95% CIs are shown, with the size of the squares proportional to study weight, and the diamond represents the pooled effect estimate from a random-effects model.

Grape seed extract showed a statistically significant (*p* < 0.05) additional PPD reduction at three months, albeit with substantial heterogeneity, suggesting inconsistent effects across studies (Figure 19A). At six months, no significant heterogeneity was detected (I^2^ = 0%) between groups (Figure 19B). CAL gain was statistically significant (*p* < 0.05) at three months (Figure 20), favoring the adjunctive therapy, but heterogeneity was present. PI outcomes showed a small but significant benefit for grape seed extract at three months (*p* < 0.05) (Figure 21A), whereas no significant heterogeneity was detected (I^2^ = 0%) at six months (Figure 21B).

**Figure 22:**
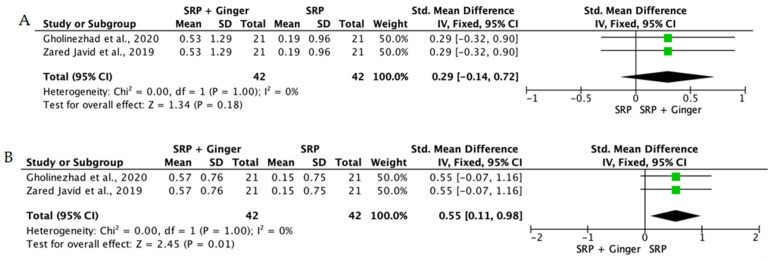
(**A**,**B**). Forest plot of the SMD in PPD (**A**) and CAL (**B**) levels at 8 weeks, respectively, comparing the SRP + Ginger with the SRP-only control group [90,92]. Individual study effect sizes with 95% CIs are shown, with the size of the squares proportional to study weight, and the diamond represents the pooled effect estimate from a random-effects model.

Finally, ginger supplementation was evaluated over an eight-week period. The pooled PPD analysis suggested a trend toward a greater reduction in the adjunctive group, although the difference was not statistically significant (95% CI crossed zero) (Figure 22A). CAL outcomes showed significantly greater CAL gain in adjunct group. No significant heterogeneity was detected I^2^ = 0%) (*p* < 0.05) (Figure 22B).

Overall, the meta-analyses indicate that several natural compounds, particularly curcumin, resveratrol, omega-3, and *Aloe vera*, provide short-term adjunctive benefits for SRP, reflected primarily in greater PPD reduction and improved inflammatory markers. These effects tend to diminish over longer follow-up periods, and heterogeneity across studies is frequent, underscoring variability in study design, formulations, dosages, and patient populations. These improvements were most consistently observed within short- to medium-term follow-up intervals, reflecting the predominant evaluation window of the included trials.

### 3.7. Certainty of Evidence

The certainty of evidence for the effects of adjunctive natural products on periodontal clinical parameters was evaluated using the GRADE framework, considering risk of bias, inconsistency, indirectness, and imprecision. Overall, the certainty of evidence ranged from high to very low, depending on the intervention, clinical outcome, and follow-up period (Table 3, Table 4 and Table 5).

For PPD (Table 3), moderate-to-high certainty evidence supported short-term benefits for several natural products. Curcumin demonstrated moderate certainty evidence for clinically relevant PPD reduction at 1 and 3 months, with large standardized mean differences favoring adjunctive therapy, while evidence at 6 months reached high certainty but showed a smaller, non-significant effect size, indicating attenuation of the benefit over time. Omega-3 supplementation showed high-certainty evidence for PPD reduction at both 1 and 3 months, with consistent, clinically meaningful effect sizes. *Aloe vera* demonstrated moderate certainty at 3 months, but certainty declined to low at 6 months due to inconsistent findings across studies. Grape seed extract exhibited high certainty evidence for PPD reduction at 3 months; however, this effect was no longer evident at 6 months, with certainty downgraded to moderate due to imprecision. Other herbal formulations showed predominantly low to moderate certainty, reflecting heterogeneity and methodological limitations.

For CAL (Table 4), the certainty of evidence followed a similar pattern. Curcumin showed moderate certainty evidence for CAL gain at 1 and 3 months, but certainty decreased to low at 6 months because of inconsistency and imprecision. Omega-3 supplementation demonstrated high certainty evidence for CAL gain at both 1 and 3 months, indicating robust and consistent adjunctive benefits. *Aloe vera* showed moderate certainty evidence for CAL gain at both 3 and 6 months, with relatively large effect sizes. Herbal formulations and grape seed extract provided moderate certainty evidence for CAL improvement at intermediate follow-up, while ginger supplementation showed high certainty evidence for CAL gain at short-term follow-up.

For BOP (Table 5), the certainty of evidence varied widely. *Aloe vera* demonstrated moderate certainty evidence for marked reductions in BOP at both 3 and 6 months, with very large effect sizes. Resveratrol showed moderate certainty evidence for BOP reduction at 1 month. In contrast, omega-3 supplementation yielded moderate to low certainty evidence, and *Ocimum sanctum* showed very low certainty evidence, largely due to serious risk of bias, inconsistency, and imprecision.

Across outcomes, downgrading of evidence certainty was most commonly driven by methodological limitations in blinding and allocation concealment, as well as by heterogeneity among studies. Nevertheless, several natural products, particularly omega-3 fatty acids, curcumin, *Aloe vera*, and grape seed extract, demonstrated moderate to high certainty evidence for short-term improvements in key periodontal parameters when used as adjuncts to SRP.

## 4. Discussion

The present systematic review and meta-analysis provide a comprehensive synthesis of current clinical evidence on the adjunctive use of natural products in NSPT. Collectively, the findings indicate that a broad range of biologically derived compounds can confer additional clinical benefits when combined with SRP, particularly in reducing PPD, improving CAL gain, and attenuating gingival inflammation. These effects were observed across diverse formulations, delivery routes, and patient populations, supporting the concept that biologically active compounds capable of modulating host–biofilm interactions may serve as valuable complementary tools in periodontal care [4,25,68].

This review extends beyond prior agent-specific syntheses by integrating a broad spectrum of natural compounds within a unified analytical framework. Whereas previous systematic reviews often focused on individual agents or narrow compound classes, the present analysis incorporates a larger and more contemporary body of randomized clinical evidence and provides quantitative evaluation of core periodontal outcomes [73,74,75,76,77,78,79,80,81,82,83]. This broader perspective enables identification of convergent therapeutic patterns across heterogeneous biologically active agents, strengthening the interpretation that host-modulatory adjunctive strategies may provide measurable clinical value.

The observed adjunctive effects are biologically plausible within the contemporary understanding of periodontitis as a dysbiosis-driven inflammatory disease sustained by maladaptive host responses [1,2,6,7,14]. While SRP remains essential for mechanical disruption of subgingival biofilms, it does not directly address the inflammatory and oxidative pathways that perpetuate connective tissue breakdown and alveolar bone loss. Many natural compounds evaluated in the included trials exhibit pleiotropic biological activities, including suppression of pro-inflammatory signaling, attenuation of oxidative stress, modulation of osteoclastogenic pathways, and interference with microbial virulence, which together provide a mechanistic framework linking host-response regulation to improved periodontal outcomes [2,23,24,51,52,65,66,67]. These molecular actions are consistent with the observed clinical improvements in PPD reduction, CAL gain, and inflammatory indices such as BOP and gingival inflammation.

Reductions in local inflammatory burden likely contribute to enhanced vascular stability, decreased immune cell infiltration, and improved tissue repair dynamics, thereby supporting more favorable post-debridement healing. Compounds such as curcumin, resveratrol, propolis, green tea catechins, *Aloe vera*, and omega-3 polyunsaturated fatty acids have repeatedly demonstrated the ability to downregulate key inflammatory mediators, including IL-1β, IL-6, TNF-α, and CRP, at both local and systemic levels [56,57,58,59,60,61,62,63,72,86,89,90,108,109,110,111,112,113,114,115,116,169]. Although most included trials were designed to evaluate clinical rather than molecular endpoints, the convergence between mechanistic rationale and measurable clinical improvements reinforces the translational relevance of adjunctive natural therapies. By integrating biological plausibility with clinical evidence, these findings illustrate how modulation of host–microbe interactions and inflammatory signaling pathways can translate into tangible periodontal benefits, supporting the relevance of clinically oriented synthesis within a broader molecular science framework [63,64,65,66,67,68,173].

In this study, local drug-delivery systems were the most frequently investigated modality [99,102,114,115,127,128,131,135,145,146,147,150,152,174,175,176] and consistently showed short-term clinical benefits, particularly in deep or persistent periodontal pockets (Table 2A). Subgingival gels, chips, nanofibers, and biodegradable carriers enable high local concentrations of active compounds while minimizing systemic exposure, making them particularly attractive for periodontal applications. Moreover, advances in formulation science, including nanoparticle-based delivery platforms and sustained-release systems, appear to enhance bioavailability and prolong therapeutic activity within the periodontal pocket. Nevertheless, substantial variability in carrier materials, dosing regimens, and application frequency across studies limits direct comparability and underscores the need for standardized delivery protocols in future clinical trials.

Systemic administration of natural products, although less frequently studied, showed notable benefits in specific clinical contexts (Table 2B) [61,177]. Resveratrol and omega-3 fatty acids, in particular, demonstrated clinically meaningful effects on both periodontal parameters and systemic inflammatory markers, especially in patients with smoking habits or metabolic comorbidities such as diabetes mellitus [26,62,89,91,112,158]. These findings align with the growing recognition of periodontitis as a disease with systemic inflammatory consequences and bidirectional links to chronic non-communicable diseases. By modulating systemic inflammation and oxidative stress, systemically administered natural compounds may exert indirect yet clinically relevant effects on periodontal tissues, supporting a more integrated approach to patient management.

The effectiveness of natural adjuncts in medically compromised populations is particularly relevant to clinical practice. Patients with diabetes [25,70,90,91,92,178], rheumatoid arthritis [171], Down syndrome [39,95], or other systemic conditions often exhibit impaired immune regulation and delayed periodontal healing, limiting the effectiveness of conventional therapy alone. Several studies included in this review demonstrated that natural products can attenuate exaggerated inflammatory responses and improve periodontal outcomes in these populations, suggesting a role for adjunctive biologically active compounds in personalized periodontal therapy [25,70,90,91,92,178]. These findings resonate with current concepts of precision medicine and host modulation in periodontology.

While the meta-analyses demonstrated statistically significant adjunctive benefits of natural products for PPD reduction and CAL gain, the magnitude of these effects warrants careful clinical interpretation. Across pooled analyses, the additional improvements beyond SRP alone were generally modest, typically within a fraction of a millimeter; however, even small incremental improvements may be clinically meaningful in specific scenarios, such as sites with residual inflammation, patients with impaired host response, or individuals at elevated risk for disease progression. Determining whether such improvements translate into meaningful long-term benefits for patients, including reduced need for surgical intervention or improved tooth survival, requires further investigation. Future studies should prioritize patient-centered outcomes, cost-effectiveness analyses, and comparisons with established adjunctive therapies to define better the clinical positioning of natural products within periodontal treatment. Importantly, periodontitis control is achieved through cumulative and sustained effects rather than single interventions. Modest adjunctive benefits may therefore contribute to improved long-term stability when integrated into comprehensive periodontal care, particularly in patients who exhibit suboptimal response to SRP alone. In this context, natural products should not be viewed as replacements for SRP, but rather as supportive measures that may help optimize outcomes in selected clinical situations.

Importantly, the clinical relevance of the observed effect sizes must be interpreted in the context of substantial clinical, methodological, and temporal heterogeneity across the included studies. Variability in compound type, formulation, dosage, delivery modality, patient characteristics, and follow-up intervals likely influenced both the magnitude and underlying mechanisms of treatment response. Differences in route of administration, ranging from locally delivered gels, chips, nanofibers, and irrigants to systemically administered supplements, introduce distinct biological dynamics, with local therapies targeting the subgingival microenvironment through high intra-pocket concentrations and systemic interventions primarily modulating host inflammatory and metabolic pathways. Additional variability arose from formulation complexity, as some investigations evaluated single bioactive compounds whereas others employed polyherbal or multi-component preparations, which may offer synergistic effects but complicate standardization and mechanistic attribution [99,102,114,115,127,128,131,135,145,146,147,150,152,174,175,176]. This clinical diversity is reflected in the moderate to high statistical heterogeneity observed across several pooled outcomes, with I^2^ values exceeding 50% in multiple comparisons. Rather than representing a methodological flaw, this variability likely reflects the inherently heterogeneous experimental conditions of the included trials, including differences in intervention characteristics, study design, sample size, risk-of-bias domains, and patient profiles such as disease severity, smoking status, systemic comorbidities, and baseline inflammatory burden. Despite these sources of heterogeneity, pooling was considered appropriate to estimate the overall adjunctive effect of natural products as a therapeutic class, rather than to establish equivalence among specific formulations.

Temporal limitations further influence interpretation. Most included trials evaluated outcomes within relatively short follow-up periods, typically 3–6 months, with limited long-term data available. Accordingly, the observed adjunctive benefits should be interpreted as short- to medium-term effects rather than evidence of sustained periodontal stability. While early improvements in inflammatory and clinical parameters are encouraging, their durability beyond the reported intervals remains uncertain. Interpretation must also consider broader methodological constraints: many studies were characterized by small sample sizes, short observation periods, and domains of potential bias identified through RoB2 assessment.

Consistent with these limitations, GRADE evaluations indicated that certainty of evidence ranged from moderate to low for several outcomes, reducing confidence in the precision and generalizability of pooled estimates. Interventions such as omega-3 fatty acids consistently achieved high-certainty evidence for both PPD reduction and CAL gain at early follow-up, supporting their role as biologically plausible and methodologically robust adjuncts to NSPT. In contrast, curcumin and *Aloe vera* showed predominantly moderate-certainty evidence, with larger short-term effects that tended to diminish over time, suggesting that their benefits may be more transient or dependent on repeated application and formulation characteristics. Importantly, the GRADE analysis reinforces that while natural products are generally safe and biologically attractive, their clinical implementation should be guided by the balance between effect magnitude and certainty of evidence, rather than statistical significance alone. Accordingly, the observed benefits should be viewed as supportive, pending confirmation through larger, standardized trials. Taken together, these factors suggest that although the observed clinical improvements are favorable, the current evidence remains insufficient to justify routine clinical adoption. Consequently, pooled findings should be interpreted as reflecting overall adjunctive trends rather than definitive superiority of specific formulations, and confirmation through well-designed, standardized, long-term randomized trials is necessary to clarify the true clinical relevance of adjunctive natural therapies.

Methodological features of the included studies also warrant caution. A subset of trials employed split-mouth or split-pocket designs, which reduce inter-individual variability but introduce statistical dependence between observations within the same participant. In the absence of detailed correlation parameters, meta-analytic pooling relied on reported summary statistics, a common practice that may overestimate precision and narrow confidence intervals. While this limitation is inherent to much of periodontal research, it underscores the importance of cautious interpretation of pooled effect sizes and highlights the need for improved reporting standards in future trials.

The interpretation of these findings must also consider the overall certainty of the available evidence. Given the presence of studies heterogeneity and variable risk-of-bias profiles across included trials, confidence in the pooled estimates is likely to range from low to moderate for several outcomes. While statistically significant effects were observed for key clinical parameters, these results should be interpreted with caution. In particular, limitations related to randomization procedures, allocation concealment, blinding, and incomplete outcome reporting were identified in a proportion of the included studies. Although many trials demonstrated acceptable internal validity, the cumulative risk-of-bias profile underscores that not all pooled outcomes can be interpreted with equal confidence. Consequently, confirmation from larger, methodologically robust randomized controlled trials are warranted.

From a translational perspective, the favorable safety profiles, relatively low costs, and broad biological activities of many natural compounds represent important advantages. Unlike systemic antibiotics, natural products are generally associated with minimal adverse effects and a negligible risk of antimicrobial resistance, making them potentially suitable for repeated or long-term use, particularly during supportive periodontal therapy [23,24,51,61,67,173]. Nevertheless, regulatory considerations, quality control, and standardization remain critical challenges that must be addressed before widespread clinical implementation can be recommended. Economic considerations are also central to evaluating the clinical applicability of adjunctive natural therapies. Many locally delivered herbal formulations and nutraceutical supplements are relatively inexpensive compared with conventional antimicrobials or technologically advanced adjunctive approaches; however, costs may vary according to formulation, frequency of application, and regional availability. Importantly, the potential economic advantages of these interventions must be weighed against the generally modest additional clinical benefits observed, and routine implementation for all patients cannot be universally justified based on the current evidence.

Patient compliance represents another key determinant of therapeutic effectiveness, particularly for systemically administered supplements or home-applied topical formulations that require sustained adherence. While in-office local drug-delivery systems may reduce compliance-related challenges, systemic or long-term regimens remain susceptible to variable patient adherence, which may partially account for heterogeneity in reported treatment outcomes. Accordingly, patient motivation, ability to adhere to prescribed regimens, and individual preferences should be carefully considered when selecting adjunctive therapies. With respect to safety, the majority of included studies reported favorable tolerability profiles for natural adjunctive therapies. This contrasts with certain conventional antimicrobial adjuncts, which may be associated with gastrointestinal disturbances, hypersensitivity reactions, or concerns related to antimicrobial resistance. Nonetheless, variability in formulation quality, dosing, and regulatory oversight underscores the need for cautious interpretation, particularly for polyherbal preparations or long-term systemic supplementation. Standardization and ongoing safety monitoring therefore, remain essential prerequisites for broader clinical adoption.

Finally, some limitations of this systematic review and meta-analysis should be considered when interpreting the findings. A substantial proportion of the included trials were small in scale, increasing the potential for imprecision and overestimation of treatment effects. In addition, considerable variability existed in intervention characteristics, including compound purity, concentration, delivery systems, dosing regimens, and duration of administration, which limits direct cross-study comparability. Follow-up periods were frequently short, with many trials evaluating outcomes over weeks or a few months, restricting conclusions regarding long-term periodontal stability and sustainability of treatment effects. Moreover, outcome assessments were conducted at heterogeneous time points; pooling the latest available data provides an estimate of maximal observed benefit but introduces temporal variability, as treatment responses may differ across follow-up intervals. Consequently, pooled estimates should be interpreted as reflecting overall adjunctive trends rather than uniform effects at specific time points.

Taken together, the current evidence supports the adjunctive use of natural products as a biologically plausible and clinically beneficial complement to SRP. By targeting both microbial and host-mediated components of periodontal disease, these agents offer a multifaceted therapeutic approach that aligns with contemporary understanding of periodontitis pathogenesis. However, while the observed adjunctive benefits are encouraging, routine clinical adoption requires confirmation through well-powered, methodologically rigorous randomized trials employing standardized formulations, consistent follow-up intervals, and transparent reporting of both clinical outcomes and adverse events. Continued research in this area has the potential to expand the therapeutic armamentarium of periodontology and to foster more personalized, host-oriented treatment strategies aimed at improving long-term clinical outcomes and quality of life for patients affected by periodontitis.

## 5. Conclusions

This systematic review and meta-analysis provide a comprehensive and updated synthesis of the clinical evidence supporting the adjunctive use of natural products in NSPT. The collective findings indicate that selected natural compounds, when used alongside SRP, can yield additional improvements in key periodontal parameters, including PPD reduction, CAL gain, and BOP control. These adjunctive benefits were most consistently observed over short- to medium-term follow-up and were supported by moderate to high certainty of evidence for specific interventions such as omega-3 polyunsaturated fatty acids, curcumin-based formulations, *Aloe vera*, and grape seed extract.

Despite these encouraging findings, the strength of the available evidence remains heterogeneous. Certainty varies according to intervention type, clinical outcome, and follow-up duration, and long-term effects remain insufficiently characterized. Methodological limitations, including small sample sizes, variability in formulations and dosing regimens, short observation periods, and domains of potential bias, reduce confidence in the precision and generalizability of pooled estimates. Accordingly, while adjunctive natural therapies demonstrate biologically plausible and clinically measurable short-term benefits, these findings should be interpreted cautiously and do not yet support universal routine adoption. Natural products should therefore be regarded as supportive adjuncts that may enhance conventional periodontal therapy rather than as standalone treatment alternatives. Their clinical integration should be guided by individualized patient needs and the strength of available evidence, particularly in scenarios where modulation of inflammatory or host-response pathways may offer added benefit. Future well-designed, adequately powered randomized trials employing standardized interventions, extended follow-up, and integration of clinical, microbiological, and mechanistic outcomes are essential to clarify long-term efficacy, optimize delivery strategies, and define the precise role of natural adjunctive therapies within evidence-based periodontal care.

## Figures and Tables

**Table 1:** Search strategies.

Advanced Searches
MEDLINE N = 2041 results ((“periodontal diseases”[MeSH Terms] OR “periodontitis”[MeSH Terms] OR “chronic periodontitis”[MeSH Terms] OR “aggressive periodontitis”[MeSH Terms] OR “periodont*”[Title/Abstract]) AND (“therapy”[MeSH Subheading] OR “therapeutics”[MeSH Terms] OR “therap*”[Title/Abstract] OR “treat*”[Title/Abstract] OR “disease management”[Title/Abstract] OR “care”[Title/Abstract] OR “periodontal debridement”[MeSH Terms] OR “subgingival curettage”[MeSH Terms] OR “dental scaling”[MeSH Terms] OR “root planing”[MeSH Terms] OR “scaling and root planing”[Title/Abstract] OR “nonsurgical periodontal treatment”[Title/Abstract] OR “nonsurgical periodontal therapy”[Title/Abstract] OR “non-surgical periodontal treatment”[Title/Abstract] OR “non-surgical periodontal therapy”[Title/Abstract] OR “subgingival instrumentation”[Title/Abstract]) AND (“plant preparations”[MeSH Terms] OR “plant extracts”[MeSH Terms] OR “plant oils”[MeSH Terms] OR “organic chemicals”[MeSH Terms] OR “botany”[MeSH Terms] OR “herbal medicine”[MeSH Terms] OR “teas, herbal”[MeSH Terms] OR “phytotherapy”[MeSH Terms])) AND (randomizedcontrolledtrial[Filter])
Cochrane Central Register of Controlled Trials (CENTRAL): N = 3019 Trials ID Search Hits #1. MeSH descriptor: [Periodontal Diseases] explode all trees 9537 #2. MeSH descriptor: [Periodontitis] explode all trees 4196 #3. MeSH descriptor: [Chronic Periodontitis] explode all trees 1088 #4. MeSH descriptor: [Aggressive Periodontitis] explode all trees 132 #5. MeSH descriptor: [Chronic Periodontitis] explode all trees 1088 #6. (periodont*):ti,ab,kw 18,233 #7. #1 OR #2 OR #3 OR #4 OR #5 OR #6 20,835 #8. MeSH descriptor: [Therapeutics] explode all trees 437,462 #9. MeSH descriptor: [Therapeutics] explode all trees 437,462 #10. MeSH descriptor: [Periodontal Debridement] explode all trees 140 #11. MeSH descriptor: [Subgingival Curettage] explode all trees 1002 #12. MeSH descriptor: [Dental Scaling] explode all trees 1529 #13. MeSH descriptor: [Root Planing] explode all trees 904 #14. (therap* OR treat* OR disease management OR care OR scaling and root planing OR nonsurgical periodontal treatment OR nonsurgical periodontal therapy OR non-surgical periodontal treatment OR non-surgical periodontal therapy OR subgingival instrumentation):ti,ab,kw 1,560,592 #15. #8 OR #9 OR #10 OR #11 OR #12 OR #13 OR #14 1,613,578 #16. MeSH descriptor: [Plant Preparations] explode all trees 15,507 #17. MeSH descriptor: [Plant Extracts] explode all trees 11,215 #18. MeSH descriptor: [Plant Oils] explode all trees 2844 #19. MeSH descriptor: [Organic Chemicals] explode all trees 311,991 #20. MeSH descriptor: [Botany] explode all trees 101 #21. MeSH descriptor: [Herbal Medicine] explode all trees 96 #22. MeSH descriptor: [Teas, Herbal] explode all trees 19 #23. MeSH descriptor: [Phytotherapy] explode all trees 5023 #24. #16 OR #17 OR #18 OR #19 OR #20 OR #21 OR #22 OR #23 324,627 #25. #7 AND #15 AND #24 3025
EMBASE: N = 4143 results (‘periodontal diseases’/exp OR ‘periodontal diseases’ OR ‘periodontitis’/exp OR ‘periodontitis’ OR ‘chronic periodontitis’/exp OR ‘chronic periodontitis’ OR ‘aggressive periodontitis’/exp OR ‘aggressive periodontitis’ OR ‘periodont*’:ti,ab,kw) AND (‘therapy’/exp OR ‘therapy’ OR ‘therapeutics’/exp OR ‘therapeutics’ OR ‘therap*’:ti,ab,kw OR ‘treat*’:ti,ab,kw OR ‘disease management’:ti,ab,kw OR ‘care’:ti,ab,kw OR ‘periodontal debridement’/exp OR ‘periodontal debridement’ OR ‘subgingival curettage’/exp OR ‘subgingival curettage’ OR ‘dental scaling’/exp OR ‘dental scaling’ OR ‘root planing’/exp OR ‘root planing’ OR ‘scaling and root planing’:ti,ab,kw OR ‘nonsurgical periodontal treatment’:ti,ab,kw OR ‘nonsurgical periodontal therapy’:ti,ab,kw OR ‘non-surgical periodontal treatment’:ti,ab,kw OR ‘non-surgical periodontal therapy’:ti,ab,kw OR ‘subgingival instrumentation’:ti,ab,kw) AND (‘plant preparations’/exp OR ‘plant preparations’ OR ‘plant extracts’/exp OR ‘plant extracts’ OR ‘plant oils’/exp OR ‘plant oils’ OR ‘organic chemicals’/exp OR ‘organic chemicals’ OR ‘botany’/exp OR ‘botany’ OR ‘herbal medicine’/exp OR ‘herbal medicine’ OR ‘teas, herbal’/exp OR ‘teas, herbal’ OR ‘phytotherapy’/exp OR ‘phytotherapy’) AND ‘randomized controlled trial’/de
Other sources and protocol registration databases: N = ? results ((“periodontal diseases” OR “chronic periodontitis” OR “aggressive periodontitis” OR “periodont*”) AND (“therap*” OR “treat*” OR “disease management” OR “care” OR “periodontal debridement” OR “subgingival curettage” OR “dental scaling” OR “root planing” OR “scaling and root planing” OR “nonsurgical periodontal treatment” OR “nonsurgical periodontal therapy” OR “non-surgical periodontal treatment” OR “non-surgical periodontal therapy” OR “subgingival instrumentation”) AND (“plant preparations” OR “plant extracts” OR “plant oils” OR “organic chemicals” OR “botany” OR “herbal medicine” OR “teas, herbal” OR “phytotherapy”))

**Table 3:** GRADE summary of findings for the effects of adjunctive natural products on PPD reduction following non-surgical periodontal therapy. The table presents the certainty of evidence for each intervention according to risk of bias, inconsistency, indirectness, and imprecision, along with pooled standardized mean differences (SMDs) and 95% confidence intervals at different follow-up periods. Overall certainty of evidence is rated as high, moderate, low, or very low according to GRADE Working Group criteria.

			Certainty Assessment					Summary of Results	
Outcomes	Participants (Studies)	Risk of Bias	Inconsistency	Indirect Evidence	Imprecision	Overall Certainty of Evidence	Intervention	Comparator	Standard Mean Difference (CI95%)
PPD (Curcumin [1 month])	356 (8 RCTs)	Serious ^ab^	Not serious	Not serious	Not serious	⨁⨁⨁◯ Moderate ^ab^	178	178	SMD 0.85 SD (0.62 to 1.07)
PPD (Curcumin [3 months])	262 (6 RCTs)	Serious ^ab^	Not serious	Not serious	Not serious	⨁⨁⨁◯ Moderate ^ab^	131	131	SMD 0.93 SD (0.67 to 1.20)
PPD (Curcumin [6 months])	132 (3 RCTs)	Not serious	Not serious	Not serious	Not serious	⨁⨁⨁⨁ High	66	66	SMD 0.19 SD (−0.15 to 0.53)
PPD (Tulsi [1 month])	90 (2 RCTs)	Serious ^ab^	Not serious	Not serious	Not serious	⨁⨁⨁◯ Moderate ^ab^	45	45	SMD 0.42 SD (−0.00 to 0.84)
PPD (Resveratrol [1 month])	124 (3 RCTs)	Serious ^b^	Not serious	Not serious	Not serious	⨁⨁⨁◯ Moderate ^b^	62	62	SMD 1.12 SD (0.74 to 1.50
PPD (*O. sanctum* [1 month])	50 (2 RCTs)	Serious ^b^	Not serious	Not serious	Serious	⨁⨁◯◯ Low ^b^	25	25	SMD 0.15 SD (−0.41 to 0.70)
PPD (Omega 3 [1 month])	120 (2 RCTs)	Not serious	Not serious	Not serious	Not serious	⨁⨁⨁⨁ High	60	60	SMD 0.52 SD (0.15 to 0.88)
PPD (Omega 3 [3 months])	150 (2 RCTs)	Not serious	Not serious	Not serious	Not serious	⨁⨁⨁⨁ High	75	75	SMD 0.64 SD (0.31 to 0.98
PPD (*Aloe vera* [3 months])	220 (4 RCTs)	Serious ^ab^	Not serious	Not serious	Not serious	⨁⨁⨁◯ Moderate	110	110	SMD 0.64 SD (0.37 to 0.92
PPD (*Aloe vera* [6 months])	120 (2 RCTs)	Serious ^b^	Serious	Not serious	Not serious	⨁⨁◯◯ Low ^b^	60	60	SMD 0.33 SD (−0.03 to 0.70)
PPD (Herbal [3 months])	146 (4 RCTs)	Serious ^b^	Serious	Not serious	Not serious	⨁⨁◯◯ Low ^b^	73	73	SMD 0.46 SD (0.13 to 0.80)
PPD (Herbal [6 months])	106 (3 RCTs)	Serious ^b^	Not serious	Not serious	Not serious	⨁⨁⨁◯ Moderate	53	53	SMD 0.27 SD (−0.12 to 0.65)
PDD (Grape seed [3 months])	192 (2 RCTs)	Not serious	Not serious	Not serious	Not serious	⨁⨁⨁⨁ High	96	96	SMD 0.92 SD (0.62 to 1.23)
PPD (Grape seed [6 months])	58 (2 RCTs)	Not serious	Not serious	Not serious	Serious	⨁⨁⨁◯ Moderate	29	29	SMD 0.00 SD (−0.51 to 0.51
PPD (Ginger [8 weeks])	84 (2 RCTs)	Not serious	Not serious	Not serious	Not serious	⨁⨁⨁⨁ High	42	42	SMD 0.29 SD (−0.14 to 0.72)

^a^ Methodological limitations related to allocation concealment, blinding processes, and incomplete outcomes. ^b^ Methodological limitations related to blinding of outcome assessment and incomplete outcomes. ⨁, ◯ these symbols show the certainty of evidence (high, moderate and low).

**Table 4:** GRADE summary of findings for the effects of adjunctive natural products on CAL gain following non-surgical periodontal therapy. Certainty of evidence was assessed across GRADE domains, and pooled effect estimates are reported as standardized mean differences (SMDs) with 95% confidence intervals for each intervention and follow-up interval.

			Certainty Assessment					Summary of Results	
Outcomes	Participants (Studies)	Risk of Bias	Inconsistency	Indirect Evidence	Imprecision	Overall Certainty of Evidence	Intervention	Comparator	Standard Mean Difference (CI95%)
CAL (Curcumin [1 month])	250 (6 RCTs)	Serious ^ab^	Not serious	Not serious	Not serious	⨁⨁⨁◯ Moderate ^ab^	125	125	SMD 0.62 SD (0.36 to 0.88)
CAL (Curcumin [3 months])	156 (4 RCTs)	Serious ^ab^	Not serious	Not serious	Not serious	⨁⨁⨁◯ Moderate ^ab^	78	78	SMD 0.28 SD (0.18 to 0.37)
CAL (Curcumin [6 months])	86 (2 RCTs)	Not serious	Serious	Not serious	Serious	⨁⨁◯◯ Low	43	43	SMD 0.07 SD (−0.36 to 0.49)
CAL (Tulsi [1 month])	90 (2 RCTs)	Serious ^ab^	Not serious	Not serious	Not serious	⨁⨁⨁◯ Moderate ^ab^	45	45	SMD 0.55 SD (0.13 to 0.98)
CAL (Resveratrol [1 month])	124 (3 RCTs)	Serious ^b^	Serious	Not serious	Not serious	⨁⨁◯◯ Low ^b^	62	62	SMD 0.23 SD (−0.13 to 0.59)
CAL (*O. sanctum* [1 month])	50 (2 RCTs)	Serious ^b^	Not serious	Not serious	Serious	⨁⨁◯◯ Low ^b^	25	25	SMD 0.08 SD (−0.048 to 0.63)
CAL (Omega 3 [1 month])	120 (2 RCTs)	Not serious	Not serious	Not serious	Not serious	⨁⨁⨁⨁ High	60	60	SMD 0.60 SD (0.23 to 0.97)
CAL (Omega 3 [3 months])	150 (2 RCTs)	Not serious	Not serious	Not serious	Not serious	⨁⨁⨁⨁ High	75	75	SMD 0.62 SD (0.29 to 0.95)
CAL (*Aloe vera* [3 months])	220 (4 RCTs)	Serious ^ab^	Not serious	Not serious	Not serious	⨁⨁⨁◯ Moderate ^ab^	110	110	SMD 0.82 SD (0.54 to 1.10)
CAL (*Aloe vera* [6 months])	120 (2 RCTs)	Serious ^b^	Not serious	Not serious	Not serious	⨁⨁⨁◯ Moderate ^b^	60	60	SMD 0.84 SD (0.47 to 1.22
CAL (Herbal [3 months])	146 (4 RCTs)	Serious ^b^	Not serious	Not serious	Not serious	⨁⨁⨁◯ Moderate ^b^	73	73	SMD 0.70 SD (0.36 to 1.04
CAL (Herbal [6 months])	106 (3 RCTs)	Serious ^b^	Not serious	Not serious	Not serious	⨁⨁⨁◯ Moderate ^b^	53	53	SMD 0.68 SD (0.28 to 1.07)
CAL (Grape seed [3 months])	192 (2 RCTs)	Not serious	Serious	Not serious	Not serious	⨁⨁⨁◯ Moderate	96	96	SMD 0.63 SD (0.34 to 0.93)
CAL (Ginger [8 weeks])	84 (2 RCTs)	Not serious	Not serious	Not serious	Not serious	⨁⨁⨁⨁ High	42	42	SMD 0.55 SD (0.11 to 0.98)

^a^ Methodological limitations related to allocation concealment, blinding processes, and incomplete outcomes. ^b^ Methodological limitations related to blinding of outcome assessment and incomplete outcomes. ⨁, ◯ these symbols show the certainty of evidence (high, moderate and low).

**Table 5:** GRADE summary of findings for the effects of adjunctive natural products on BOP following non-surgical periodontal therapy. Certainty of evidence was assessed across GRADE domains, and pooled effect estimates are reported as standardized mean differences (SMDs) with 95% confidence intervals for each intervention and follow-up interval.

			Certainty Assessment					Summary of Results	
Outcomes	Participants (Studies)	Risk of Bias	Inconsistency	Indirect Evidence	Imprecision	Overall Certainty of Evidence	Intervention	Comparator	Standard Mean Difference (CI95%)
BOP (Resveratrol [1 month])	124 (3 RCTs)	Serious ^b^	Not serious	Not serious	Not serious	⨁⨁⨁◯ Moderate ^b^	40	40	SMD 0.95 SD (0.48 to 1.41)
BOP (*O. sanctum* [1 month])	50 (2 RCTs)	Serious ^b^	Serious	Serious	Serious	⨁◯◯◯ Very low ^b^	25	25	SMD 0.02 SD (−0.53 to 0.58)
BOP (Omega 3 [1 month])	120 (2 RCTs)	Not serious	Not serious	Not serious	Serious	⨁⨁⨁◯ Moderate	60	60	SMD 0.25 SD (−0.11 to 0.61)
BOP (Omega 3 [3 months])	120 (2 RCTs)	Not serious	Serious	Not serious	Serious	⨁⨁◯◯ Low	60	60	SMD 0.28 SD (−0.08 to 0.64)
BOP (*Aloe vera* [3 months])	160 (2 RCTs)	Serious ^ab^	Not serious	Not serious	Not serious	⨁⨁⨁◯ Moderate ^ab^	80	80	SMD 2.11 SD (1.72 to 2.50)
BOP (*Aloe vera* [6 months])	160 (2 RCTs)	Serious ^ab^	Not serious	Not serious	Not serious	⨁⨁⨁◯ Moderate ^ab^	80	80	SMD 2.99 SD (2.53 to 3.45)

^a^ Methodological limitations related to allocation concealment, blinding processes, and incomplete outcomes. ^b^ Methodological limitations related to blinding of outcome assessment and incomplete outcomes. ⨁, ◯ these symbols show the certainty of evidence (high, moderate and low).

## Data Availability

No new data were created or analyzed in this study. Data sharing is not applicable to this article.
